# 660 nm LED preconditioning programs mitochondrial signaling in BMSCs for functional tracheal cartilage regeneration

**DOI:** 10.1016/j.mtbio.2026.103729

**Published:** 2026-10-06

**Authors:** Zhao An, Jiajun Zhang, Chao Wang, Yunlang She, Yong Xu, Haichao Zhou, Jiang Xia, Liang Guo, Minjie Ma, Lei Zhang

**Affiliations:** a Department of Thoracic Surgery, Shanghai Pulmonary Hospital, Tongji University School of Medicine, Shanghai, China; b Department of Cardiovascular Surgery, Changhai Hospital, Shanghai, China; c Department of Thoracic Surgery, The First Hospital of Lanzhou University, Lanzhou, China; d Department of Orthopedics, Tongji Hospital, School of Medicine, Tongji University School of Medicine, Shanghai, China

**Keywords:** Photonic mitochondrial programming, 660 nm LED irradiation, Photobiomodulation, Chondrogenesis, Mitochondrial ROS signaling

## Abstract

Stem-cell-based cartilage regeneration is limited not only by cell delivery, but also by the difficulty of establishing a stable and functionally competent regenerative cell state before implantation. Here, we report a photonic mitochondrial programming strategy in which 660 nm light-emitting diode (LED) irradiation preconditions bone marrow-derived mesenchymal stem cells (BMSCs) to enhance functional tracheal cartilage regeneration. BMSCs were seeded onto egg white-derived ring-shaped porous scaffolds and exposed to defined 660 nm LED irradiation before implantation. Among the tested irradiation conditions, 15 J/cm^2^ optimally promoted BMSC proliferation, chondrogenic differentiation and cartilage-like extracellular matrix deposition, while suppressing fibrotic, hypertrophic and matrix-degrading phenotypes. Mechanistically, 660 nm LED irradiation enhanced cytochrome *c* oxidase (CCO) activity, mitochondrial membrane potential, adenosine triphosphate (ATP) production and respiratory function, accompanied by moderate reactive oxygen species (ROS) signaling. This mitochondrial bioenergetic–redox response activated the PI3K/Akt/mTOR/GSK3β–SOX9 axis, thereby promoting type II collagen, aggrecan and glycosaminoglycan-rich matrix formation. Pharmacological inhibition of ROS signaling, mitochondrial activity, PI3K or mTOR attenuated the LED-induced chondrogenic program. In a rabbit tracheal cartilage ring defect model, 15 J/cm^2^-preconditioned BMSC–scaffold constructs improved cartilage-like tissue formation, airway epithelial repair, lumen patency and mechanical support after 30 days of observation. These findings support 660 nm LED irradiation as a controllable mitochondrial preconditioning strategy for stem-cell-based functional cartilage regeneration.

## Highlights

• 660 nm LED preconditioning programs the mitochondrial state of BMSCs.
• A 15 J/cm^2^ dose optimally promotes stable chondrogenic differentiation.
• Moderate ROS activates the PI3K/Akt/mTOR/GSK3β–SOX9 signaling axis.
• LED preconditioning limits fibrotic, hypertrophic and catabolic phenotypes.
• BMSC-loaded ring scaffolds restore tracheal cartilage and mechanical support.

## Graphical abstract

**Figure:**
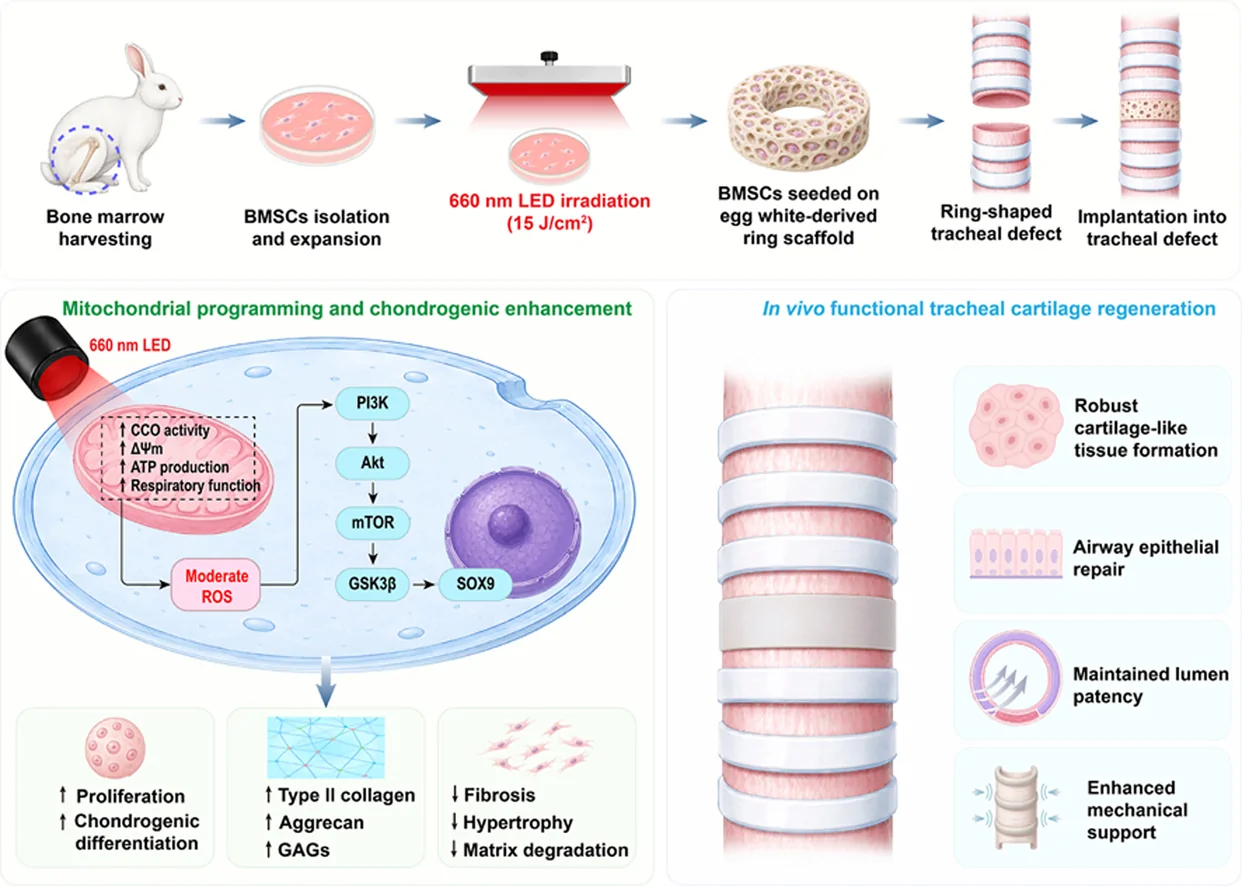

## 1 Introduction

A major limitation in stem-cell-based regeneration is the difficulty of establishing a durable and functionally competent cell state before implantation [1]. In many tissue-engineering strategies, cells are delivered into defects with the expectation that scaffold architecture, soluble factors or host microenvironmental cues will guide tissue formation [2]. However, implanted cells may remain metabolically unprepared, incompletely lineage-committed or vulnerable to inflammatory and mechanical stress [3]. This problem is particularly evident in cartilage regeneration, where successful repair requires not only cell survival and extracellular matrix deposition, but also stable activation of a chondrogenic program and suppression of fibrotic, hypertrophic and catabolic remodeling [4,5].

Current approaches to induce chondrogenesis largely depend on biochemical factors such as transforming growth factor-β (TGF-β) [6]. Although these cues can activate chondrogenic signaling, they provide limited control over the intracellular metabolic state that supports durable matrix biosynthesis [7]. Emerging evidence has shifted the view of mitochondria from passive energy-producing organelles to active regulators of stem-cell fate. Mitochondrial membrane potential, respiratory activity, adenosine triphosphate (ATP) availability and redox tone collectively define whether a stem cell is simply viable or truly competent for regeneration [8]. In this context, mitochondrial reactive oxygen species (ROS) are not merely damaging by-products, but can function as signaling mediators when maintained within a controlled range [9]. Thus, the mitochondrial bioenergetic–redox state represents a potential intracellular checkpoint for engineering stem-cell differentiation.

Red-light photobiomodulation provides a non-invasive physical route to access this mitochondrial checkpoint [10]. Light around 660 nm can be delivered using simple light-emitting diode (LED) systems and is associated with mitochondrial photoacceptors, particularly cytochrome *c* oxidase (CCO)/mitochondrial complex IV, which links photon input to respiratory chain activity, ATP production and redox signaling [11,12]. Compared with soluble biochemical stimulation, light can be applied externally, withdrawn immediately and spatially controlled without leaving residual reagents in engineered constructs [13]. These properties make 660 nm LED irradiation an attractive candidate for mitochondrial preconditioning. Yet a key question remains unresolved: can a defined red-light input write a pro-chondrogenic mitochondrial state into bone marrow-derived mesenchymal stem cells (BMSCs) that is strong enough to support functional tissue repair after implantation?

BMSC chondrogenesis offers a stringent model to address this question [14]. A successful chondrogenic state requires activation of SRY-box transcription factor 9 (SOX9) and production of type II collagen alpha 1 chain (COL2A1)-, aggrecan (ACAN)- and glycosaminoglycan (GAG)-rich matrix, while limiting type I collagen alpha 1 chain (COL1A1)-associated fibrotic matrix formation, type X collagen alpha 1 chain (COL10A1)/runt-related transcription factor 2 (RUNX2)-associated hypertrophic differentiation and matrix metalloproteinase 13 (MMP13)-mediated matrix degradation [15]. Therefore, simply increasing cell proliferation or bulk matrix deposition is insufficient. A useful preconditioning strategy must coordinate mitochondrial activation, redox signaling and chondrogenic transcription while preserving phenotypic stability.

Tracheal cartilage repair provides a demanding functional test for such a strategy. The trachea relies on cartilage rings to maintain airway patency and resist collapse during respiration [16]. Loss of a native cartilage ring creates a defect in both tissue identity and mechanical architecture [17]. Repair therefore requires regeneration of cartilage-like matrix, restoration of ring-shaped support, epithelial healing and functional resistance to stenosis or collapse [18]. A BMSC-loaded ring-shaped porous scaffold thus provides a functionally relevant platform to determine whether red-light-induced mitochondrial preconditioning can be translated from cellular fate regulation to airway cartilage reconstruction [19].

Here, we sought to address whether a defined photonic stimulus could establish a regenerative state in BMSCs before implantation and improve functional tracheal cartilage regeneration. Specifically, we investigated whether 660 nm LED preconditioning could regulate BMSC behavior through mitochondrial metabolic modulation and explored the underlying intracellular signaling mechanisms. Furthermore, we evaluated whether LED-preconditioned BMSC-loaded scaffolds could enhance cartilage reconstruction and functional recovery in a tracheal cartilage defect model. Through these studies, we aimed to establish a photonic mitochondrial preconditioning strategy for stem-cell-based functional cartilage regeneration, as illustrated in Scheme 1.

**Scheme 1:**
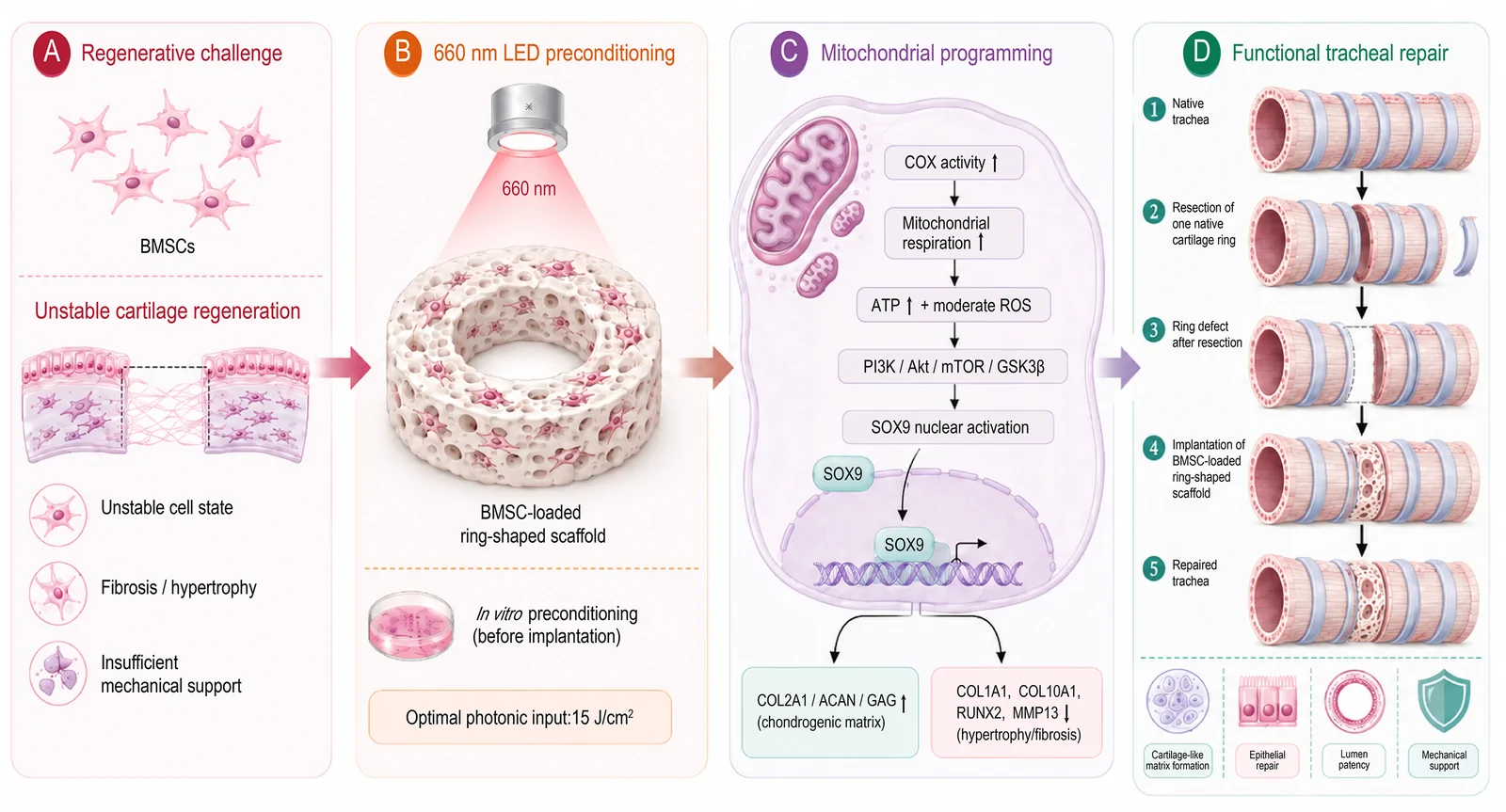
| Photonic mitochondrial programming of BMSCs for functional tracheal cartilage regeneration. (A) BMSCs were combined with a ring-shaped porous scaffold to generate a BMSC-loaded construct. (B) The construct was preconditioned with 660 nm LED irradiation before implantation. (C) LED preconditioning enhanced mitochondrial bioenergetic–redox activity, including CCO activation, mitochondrial respiration, ATP production and moderate ROS signaling, thereby activating the PI3K/Akt/mTOR/GSK3β–SOX9 axis. This response promoted COL2A1/ACAN/GAG-rich cartilage matrix formation while suppressing fibrotic, hypertrophic and matrix-degrading phenotypes. (D) The preconditioned BMSC-loaded ring-shaped scaffold was surgically implanted to replace a resected native tracheal cartilage ring, resulting in improved cartilage-like tissue regeneration, airway epithelial repair, lumen patency and mechanical support.

## 2 Results

### 2.1 660 nm LED irradiation promotes BMSC proliferation in a dose-dependent manner

Before evaluating the biological effects of 660 nm LED irradiation, BMSCs were first identified by morphology, surface markers and multilineage differentiation assays. Passage 3 BMSCs displayed a typical spindle-like fibroblastic morphology (Fig. S1A) and showed positive crystal violet staining (Fig. S1B). The marker panel was selected according to the International Society for Cell & Gene Therapy (ISCT) minimal criteria for mesenchymal stem cells (MSC) characterization and previous rabbit MSC studies, with consideration of species-specific variability in MSC surface marker expression [20]. Flow cytometry confirmed high expression of mesenchymal-associated markers CD29, CD44, CD90 and CD105, together with low expression of hematopoietic markers CD34 and CD45 (Fig. S1C and D), supporting the identity of the isolated rabbit BMSCs. The cells also exhibited adipogenic, osteogenic and chondrogenic differentiation potential, as demonstrated by Oil Red O, Alizarin Red and Alcian blue staining, respectively (Fig. S1E and F). These results confirmed that the isolated cells met the basic phenotypic criteria for BMSCs.

We next established a 660 nm LED irradiation system for BMSC treatment (Fig. 1A). To exclude nonspecific thermal effects, culture medium temperature was monitored during 0–10 min irradiation (Fig. S2A). The maximum temperature elevation increased with irradiation dose, and the temperature control (TC) group was matched to the 30 J/cm^2^ LED group without light exposure (Fig. S2B). This design allowed us to distinguish the biological effects of 660 nm photobiomodulation from those caused by mild temperature changes.

**Fig. 1:**
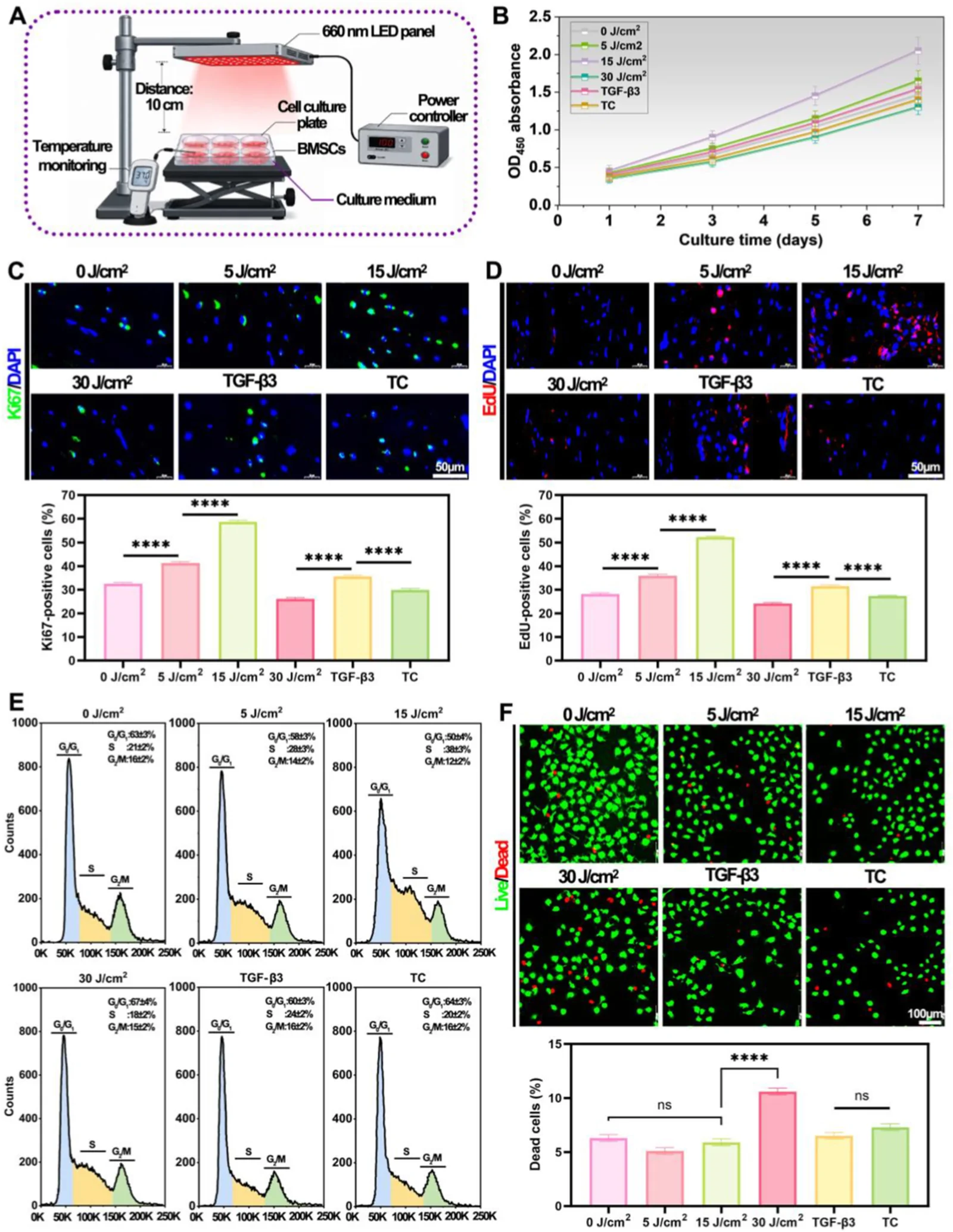
| Dose-dependent effects of 660 nm LED irradiation on BMSC viability and proliferation. **(A)** Schematic illustration for 660 nm LED irradiation of BMSCs. **(B)** CCK-8 analysis of BMSC proliferation from day 1 to day 7 after treatment with 0, 5, 15 or 30 J/cm^2^ 660 nm LED irradiation, TGF-β3 or temperature control (TC). The 0 J/cm^2^ group served as the untreated control; TGF-β3 was included as a biochemical chondrogenic reference control rather than a proliferation-positive control, and the TC group was temperature-matched to the 30 J/cm^2^ LED group without light exposure. **(C)** Representative Ki67 immunofluorescence images and quantification of Ki67-positive cells at day 3. **(D)** Representative EdU staining images and quantification of EdU-positive cells at day 3. **(E)** Flow cytometric analysis and quantification of cell-cycle distribution in each group at day 3. **(F)** Representative Live/Dead staining images and quantification of dead-cell percentage in each group at day 3, showing cell viability. Data are presented as mean ± SEM; n = 5 biologically independent samples. Statistical significance was determined by two-way ANOVA with Tukey's multiple-comparison test for B and one-way ANOVA with Tukey's multiple-comparison test for C–F. ns, not significant; ****P < 0.0001.

Cell Counting Kit-8 (CCK-8) analysis showed that 660 nm LED irradiation regulated BMSC proliferation in a dose-dependent manner from day 1 to day 7 (Fig. 1B). The time-course analysis demonstrated that the proliferative response to LED stimulation was dynamically regulated over time. Among the tested doses, 15 J/cm^2^ induced the most sustained and pronounced increase in cell proliferation throughout the observation period, whereas 5 J/cm^2^ showed a moderate effect and 30 J/cm^2^ failed to further enhance proliferation and exhibited reduced proliferative activity compared with the 15 J/cm^2^ group, suggesting a biphasic dose-response pattern. Ki67 immunofluorescence showed increased proliferative activity after 15 J/cm^2^ irradiation at day 3 (Fig. 1C). EdU staining further confirmed that the 15 J/cm^2^ group had a higher proportion of DNA-synthesizing cells than the 0 J/cm^2^ and TC groups (Fig. 1D). Importantly, the TC group, which was temperature-matched to the 30 J/cm^2^ LED group without light exposure, did not show the reduced proliferative activity observed in the 30 J/cm^2^ LED group, indicating that the inhibitory effect associated with high-dose LED irradiation was unlikely to be primarily caused by thermal effects.

Flow cytometric cell-cycle analysis revealed that 15 J/cm^2^ irradiation increased the proportion of cells in S phase, indicating enhanced cell-cycle progression (Fig. 1E and Fig. S3A and B). Live/Dead staining showed that 15 J/cm^2^ irradiation maintained high cell viability, whereas 30 J/cm^2^ did not provide additional proliferative benefit and showed a tendency toward reduced cellular activity (Fig. 1F). Collectively, these data indicate that 660 nm LED irradiation exerts a biphasic dose-dependent effect on BMSC proliferation, with 15 J/cm^2^ representing the optimal dose for promoting cell proliferation without obvious cytotoxicity.

### 2.2 15 J/cm^2^ 660 nm LED irradiation optimally enhances BMSC chondrogenic differentiation

To determine whether 660 nm LED irradiation also regulates BMSC chondrogenesis, BMSCs were induced to form chondrogenic pellets and treated with different irradiation doses according to the experimental timeline (Fig. 2A). At day 14, gross observation showed that pellets in the 15 J/cm^2^ group were larger, more regular and more compact than those in the 0 J/cm^2^, 30 J/cm^2^ and TC groups (Fig. 2B). Quantitative analysis further confirmed increased pellet surface area, wet weight and compactness score in the 15 J/cm^2^ group (Fig. 2C).

**Fig. 2:**
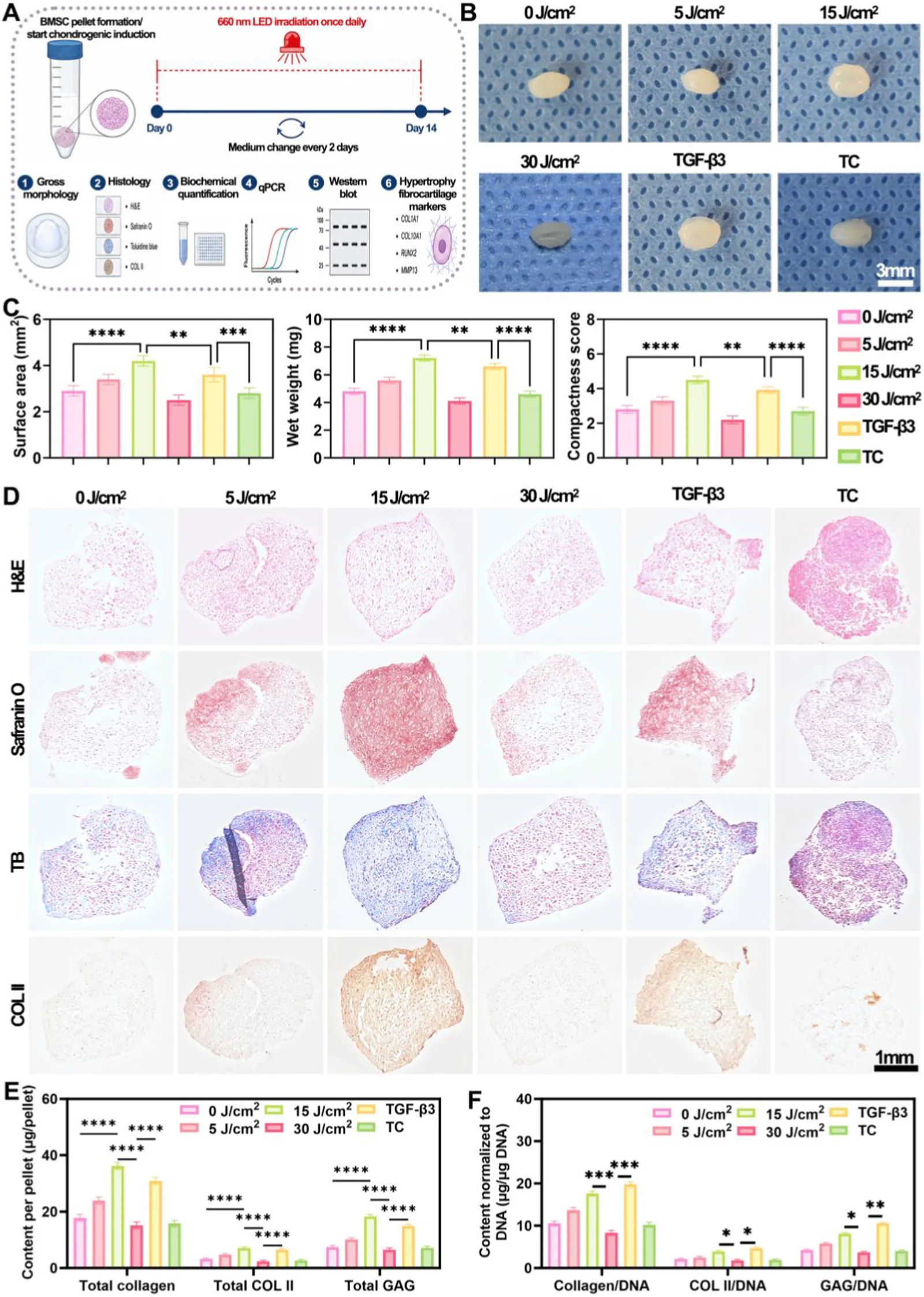
| 15 J/cm^2^ 660 nm LED irradiation optimally enhances chondrogenic differentiation of BMSCs. **(A)** Experimental timeline of BMSC chondrogenic induction with 660 nm LED irradiation. BMSCs were induced to form cartilage pellets from day 0 and treated with 0, 5, 15 or 30 J/cm^2^ 660 nm LED irradiation, TGF-β3 or TC. Samples were collected at day 14 for gross morphology, histological staining, biochemical assays, RT-qPCR, western blotting, and analysis of chondrogenic, hypertrophic, fibrotic and matrix-degrading markers. **(B)** Representative gross images of BMSC pellets in various groups at day 14. **(C)** Quantification of pellet surface area, wet weight and compactness score at day 14. **(D)** Representative H&E, Safranin O, Toluidine blue (TB) and COL II immunohistochemical staining images of BMSC pellets at day 14. **(E)** Biochemical quantification of total collagen, total COL II and total GAG contents in BMSC pellets after 14 days of chondrogenic induction. **(F)** DNA-normalized matrix production, including collagen/DNA, COL II/DNA and GAG/DNA ratios. Data are presented as mean ± SEM; n = 5 biologically independent samples. One-way ANOVA followed by Tukey's test was used for surface area and wet weight, whereas compactness scores were analyzed using the Kruskal–Wallis test followed by Dunn's multiple-comparison test. ns, not significant; *P < 0.05, **P < 0.01, ***P < 0.001, ****P < 0.0001. (For interpretation of the references to colour in this figure legend, the reader is referred to the Web version of this article.)

Histological staining supported the gross morphological findings. H&E staining showed denser tissue organization in the 15 J/cm^2^ group, while Safranin O and Toluidine blue (TB) staining demonstrated stronger proteoglycan deposition (Fig. 2D and Fig. S4). COL II immunohistochemistry further revealed enhanced cartilage-specific matrix accumulation after 15 J/cm^2^ irradiation (Fig. 2D and Fig. S4). Biochemical assays confirmed that total collagen, total COL II and total GAG contents were increased in the 15 J/cm^2^ group compared with the 0 J/cm^2^ and TC groups (Fig. 2E). After normalization to DNA content, collagen/DNA, COL II/DNA and GAG/DNA ratios remained highest in the 15 J/cm^2^ group (Fig. 2F), indicating that 15 J/cm^2^ irradiation enhanced matrix production per cell rather than merely increasing cell number.

At the protein level, western blotting showed increased expression of SOX9, COL II and ACAN in the 15 J/cm^2^ group (Fig. 3A and B). These molecules represent key regulators and matrix components of chondrogenic differentiation. In contrast, immunofluorescence staining showed that fibrotic, hypertrophic and matrix-degrading markers, including COL1A1, COL10A1, RUNX2 and MMP13, were reduced after 15 J/cm^2^ irradiation (Fig. 3C). Western blotting and quantitative analysis further confirmed decreased expression of these undesirable markers in the 15 J/cm^2^ group (Fig. 3D and E). Reverse transcription quantitative polymerase chain reaction (RT-qPCR) analysis showed a similar trend at the mRNA level, with lower *Col1a1*, *Col10a1*, *Runx2* and *Mmp13* expression in the 15 J/cm^2^ group than in the 30 J/cm^2^ and TC groups (Fig. 3F).

**Fig. 3:**
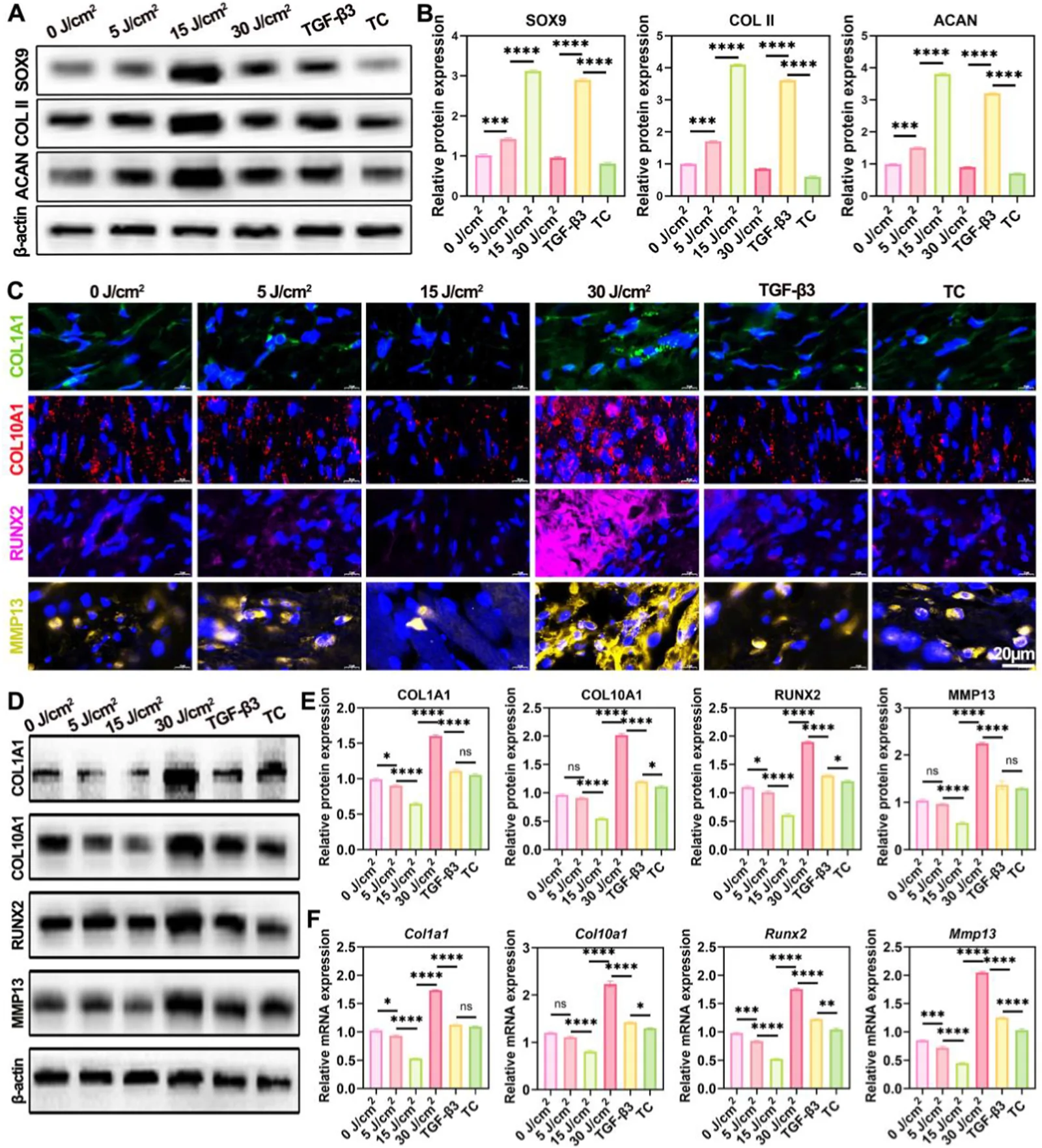
| 15 J/cm^2^ 660 nm LED irradiation promotes chondrogenic protein expression and suppresses fibrotic, hypertrophic and matrix-degrading markers in BMSC pellets. **(A)** Representative western blot images of SOX9, COL II and ACAN in BMSC pellets at day 14 after treatment with 0, 5, 15 or 30 J/cm^2^ 660 nm LED irradiation, TGF-β3 or TC. **(B)** Quantification of SOX9, COL II and ACAN protein expression in the indicated groups. **(C)** Representative immunofluorescence staining images of COL1A1, COL10A1, RUNX2 and MMP13 in BMSC pellets at day 14. **(D)** Representative western blot images of COL1A1, COL10A1, RUNX2 and MMP13. **(E)** Quantitative analysis of COL1A1, COL10A1, RUNX2 and MMP13 protein expression. **(F)** RT-qPCR analysis of *Col1a1*, *Col10a1*, *Runx2* and *Mmp1*3 mRNA expression. BMSC pellets were treated with 0, 5, 15 or 30 J/cm^2^ 660 nm LED irradiation, TGF-β3 or TC. Western blot data were normalized to β-actin and expressed relative to the 0 J/cm^2^ group. Data are presented as mean ± SEM; n = 5 biologically independent samples. Statistical significance was determined by one-way ANOVA with Tukey's multiple-comparison test. ns, not significant; *P < 0.05, **P < 0.01, ***P < 0.001, ****P < 0.0001.

Together, these results demonstrate that 15 J/cm^2^ 660 nm LED irradiation enhances BMSC chondrogenic differentiation and promotes cartilage-related matrix formation while reducing fibrotic and hypertrophic marker expression.

### 2.3 LED pretreatment improves BMSC behavior and cartilage-like matrix formation in ring-shaped porous scaffolds

To translate the optimized LED treatment into a tissue-engineering strategy, egg white-derived biomaterial was fabricated into a ring-shaped porous scaffold, seeded with BMSCs and subjected to 660 nm LED irradiation (Fig. 4A). Gross and scanning electron microscope (SEM) images confirmed that the scaffold had a ring-shaped structure and a porous microarchitecture suitable for cell loading and tissue ingrowth (Fig. 4B).

**Fig. 4:**
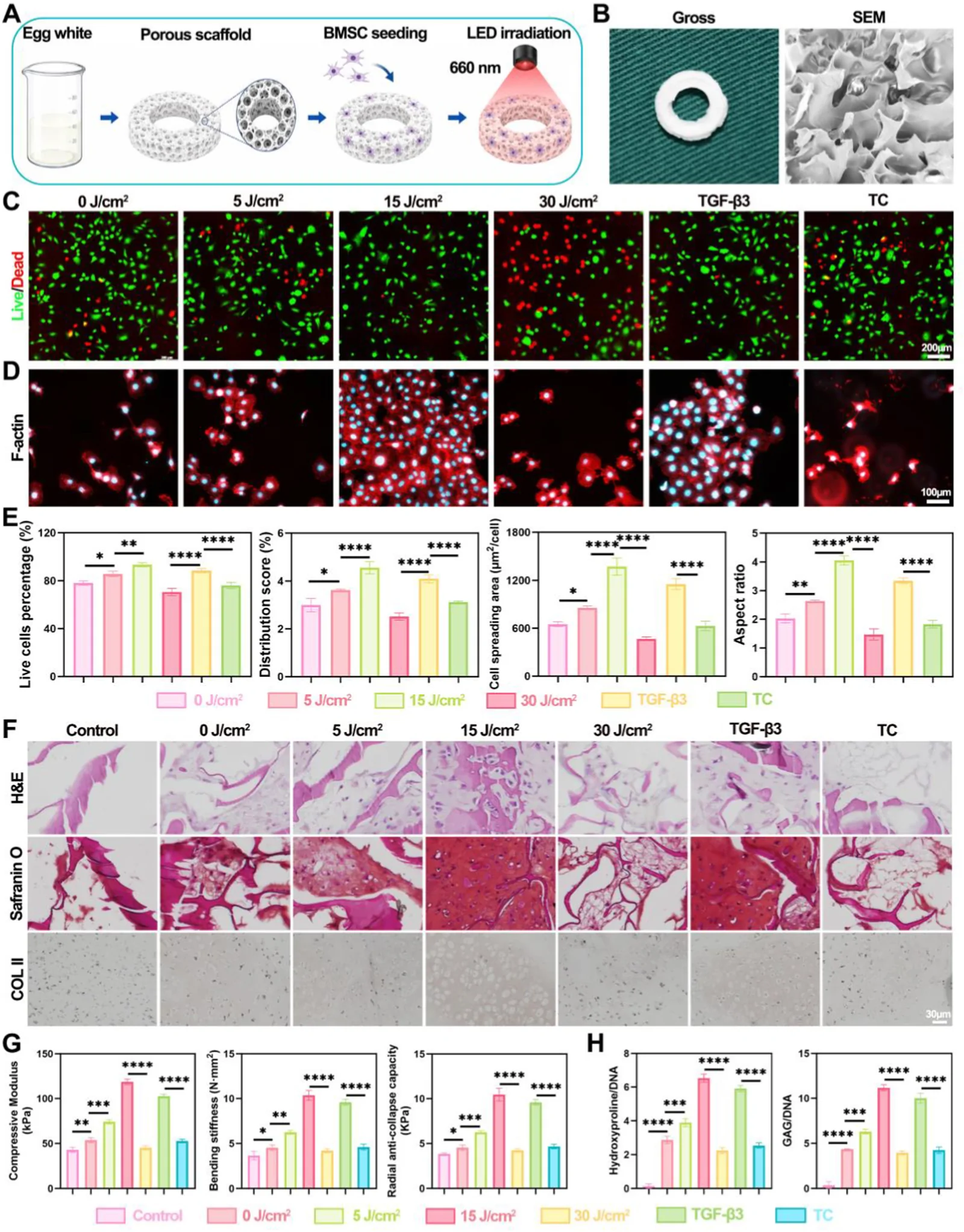
| 15 J/cm^2^ 660 nm LED pretreatment promotes cartilage-like matrix formation in BMSC-loaded porous scaffolds. **(A)** Schematic illustration of the preparation and 660 nm LED pretreatment of BMSC-loaded ring-shaped porous scaffolds. Egg white-derived biomaterial was fabricated into a ring-shaped porous scaffold, seeded with BMSCs and subsequently subjected to 660 nm LED irradiation. **(B)** Gross appearance and scanning electron microscopy (SEM) images of the ring-shaped porous scaffold. **(C)** Representative Live/Dead staining images of BMSC-seeded scaffolds at day 3 after treatment with 0, 5, 15 or 30 J/cm^2^ 660 nm LED irradiation, TGF-β3 or TC. **(D)** Representative F-actin staining images showing BMSC morphology and spreading within scaffolds at day 3. **(E)** Quantification of live-cell percentage, cell distribution score, cell spreading area and aspect ratio in BMSC-seeded scaffolds at day 3. **(F)** Representative images of H&E staining, Safranin O staining, and COL II immunohistochemical staining of constructs from the Control, 0, 5, 15, and 30 J/cm^2^, TGF-β3, and TC groups on day 14. **(G)** Quantification of compressive modulus, bending stiffness and radial anti-collapse capacity of constructs from each group at day 14. **(H)** Quantification of hydroxyproline/DNA and GAG/DNA levels in constructs at day 14. The Control group indicates acellular porous scaffolds without BMSC seeding. The 0 J/cm^2^ group served as the untreated BMSC-scaffold control; TGF-β3 was used as a positive chondrogenic control, and the TC group was temperature-matched to the 30 J/cm^2^ LED group without light exposure. Data are presented as mean ± SEM; n = 5 biologically independent samples. Statistical significance was determined by one-way ANOVA with Tukey's multiple-comparison test for E, G and H. *P < 0.05, **P < 0.01, ***P < 0.001, ****P < 0.0001.

At day 3, Live/Dead staining demonstrated that BMSCs remained viable within the scaffolds after LED irradiation (Fig. 4C). The 15 J/cm^2^ group showed a higher live-cell percentage and more uniform cell distribution than the 0 J/cm^2^ and TC groups. F-actin staining further showed improved cell spreading and cytoskeletal organization in the 15 J/cm^2^ group (Fig. 4D). Quantitative analysis confirmed increased cell distribution score, cell spreading area and aspect ratio after 15 J/cm^2^ irradiation (Fig. 4E). These results suggest that 660 nm LED pretreatment improves early BMSC survival, distribution and adhesion within the porous scaffold microenvironment.

At day 14, histological analysis showed more abundant tissue formation and cartilage-like matrix deposition in 15 J/cm^2^-pretreated BMSC-scaffold constructs (Fig. 4F). H&E staining revealed improved tissue filling within scaffold pores, while Safranin O and COL II immunohistochemistry showed stronger GAG and type II collagen deposition (Fig. 4F). Mechanical testing demonstrated that 15 J/cm^2^ pretreatment increased compressive modulus, bending stiffness and radial anti-collapse capacity compared with untreated BMSC-scaffold constructs and TC controls (Fig. 4G). Biochemical analysis further showed increased hydroxyproline/DNA and GAG/DNA levels in the 15 J/cm^2^ group (Fig. 4H). To further exclude the possibility that the enhanced tissue formation within the scaffold constructs was associated with osteogenic matrix formation, Osteocalcin (OCN) immunofluorescence staining was performed using the same BMSC-loaded porous scaffold system (Fig. S5A). The results showed that 15 J/cm^2^ LED pretreatment did not increase OCN expression compared with the control and TGF-β3 groups, whereas the 30 J/cm^2^ irradiation group exhibited elevated OCN fluorescence intensity. Quantitative analysis further confirmed that optimized 15 J/cm^2^ LED pretreatment did not promote the expression of osteogenic markers (Fig. S5B). These results demonstrate that 660 nm LED pretreatment enhances BMSC survival, distribution, matrix formation, and mechanical properties of BMSC-loaded porous scaffolds.

### 2.4 15 J/cm^2^ irradiation enhances mitochondrial function and induces moderate ROS signaling during chondrogenesis

Because red-light photobiomodulation is closely associated with mitochondrial regulation [21], we next examined mitochondrial function during BMSC chondrogenesis. JC-1 staining showed stronger red fluorescence and weaker green fluorescence in the 15 J/cm^2^ group (Fig. 5A), indicating increased mitochondrial membrane potential [22]. Quantification of the JC-1 red/green fluorescence ratio confirmed that 15 J/cm^2^ irradiation significantly enhanced mitochondrial membrane potential compared with the 0 J/cm^2^ and TC groups (Fig. 5B).

**Fig. 5:**
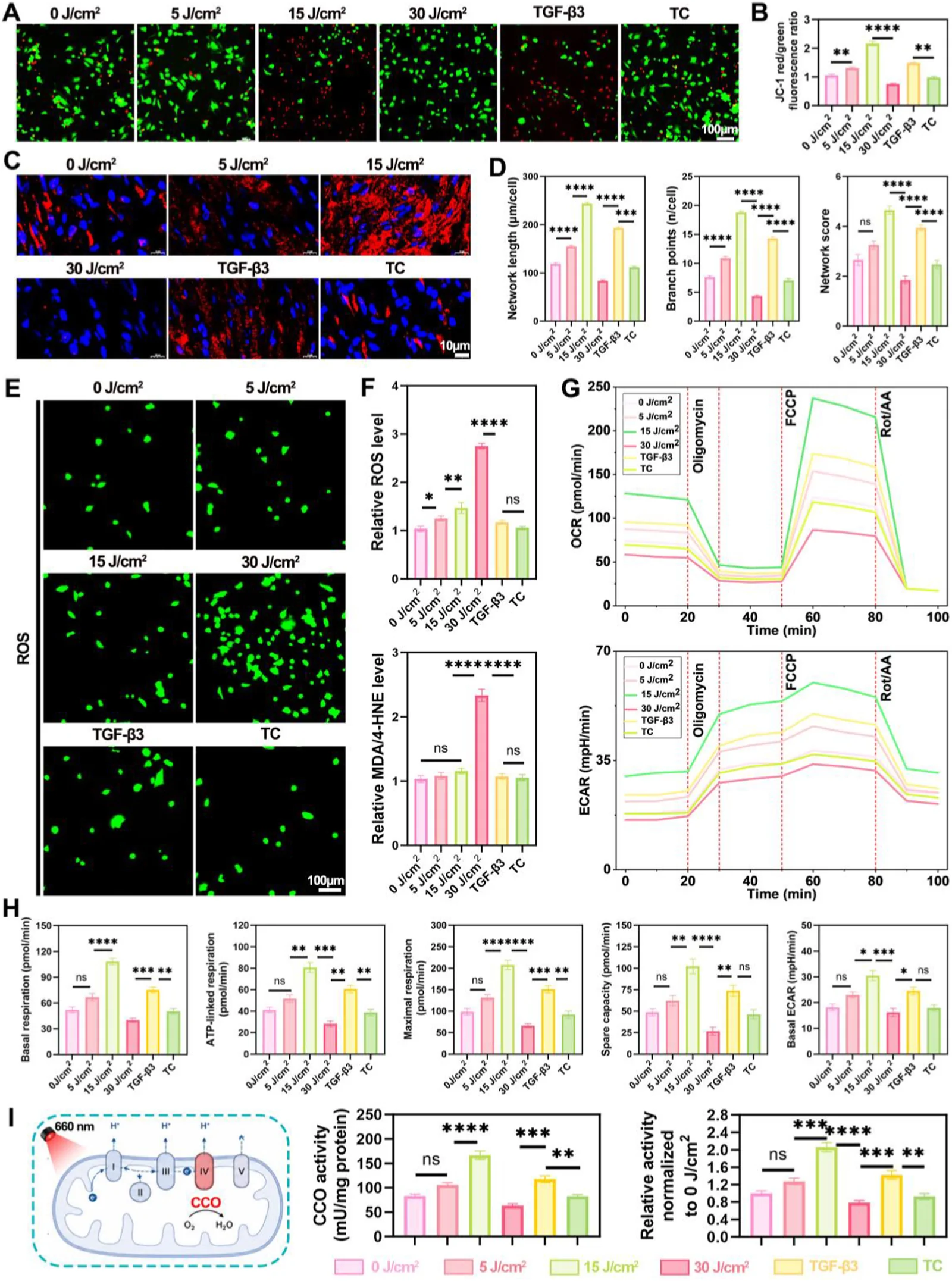
| 15 J/cm^2^ 660 nm LED irradiation enhances mitochondrial function during BMSC chondrogenesis. **(A)** Representative JC-1 staining images showing mitochondrial membrane potential in BMSC pellets at day 14 after treatment with 0, 5, 15 or 30 J/cm^2^ 660 nm LED irradiation, TGF-β3 or TC. **(B)** Quantification of the JC-1 red/green fluorescence ratio. **(C)** Representative MitoTracker Red staining images showing mitochondrial network morphology at day 14. Nuclei were counterstained with DAPI. **(D)** Quantification of mitochondrial network length, branch points and network score. **(E)** Representative MitoPY1 staining images showing mitochondrial H_2_O_2_/ROS levels in BMSCs after treatment with different doses of 660 nm LED irradiation. Green fluorescence indicates mitochondrial H_2_O_2_ signal. **(F)** Quantification of MitoPY1 fluorescence intensity and oxidative stress markers, including MDA and 4-HNE levels. **(G)** Oxygen consumption rate (OCR) and extracellular acidification rate (ECAR) were measured using a Seahorse extracellular flux analyzer at day 14. All OCR and ECAR values were normalized to total protein content per well. **(H)** Quantification of basal respiration, ATP-linked respiration, maximal respiration, spare respiratory capacity and basal ECAR. **(I)** Schematic illustration and quantification of CCO activity, reflecting mitochondrial respiratory chain complex IV function. CCO activity was normalized to the 0 J/cm^2^ group. Data are presented as mean ± SEM; n = 5 biologically independent samples. Statistical significance was determined by two-way ANOVA with Tukey's multiple-comparison test for G and one-way ANOVA with Tukey's multiple-comparison test for B, D, F, H and I. ns, not significant; *P < 0.05, **P < 0.01, ***P < 0.001, ****P < 0.0001. (For interpretation of the references to colour in this figure legend, the reader is referred to the Web version of this article.)

MitoTracker staining revealed a more interconnected mitochondrial network in the 15 J/cm^2^ group (Fig. 5C). Quantitative analysis showed increased mitochondrial network length, branch points and network score after 15 J/cm^2^ irradiation (Fig. 5D). Consistently, ATP production was elevated in the 15 J/cm^2^ group, as shown by increased ATP content and relative ATP level normalized to the 0 J/cm^2^ group (Fig. S6).

Mitochondrial H_2_O_2_/ROS analysis revealed a dose-dependent pattern. MitoPY1 staining showed that 15 J/cm^2^ irradiation induced a moderate increase in mitochondrial H_2_O_2_ signal (Fig. 5E). In contrast, 30 J/cm^2^ irradiation produced a higher MitoPY1 fluorescence signal and increased malondialdehyde (MDA) and 4-hydroxynonenal (4-HNE) levels, indicating excessive oxidative stress and lipid peroxidation (Fig. 5F). These results suggest that moderate mitochondrial H_2_O_2_ signaling induced by 15 J/cm^2^ irradiation may function as a signaling mediator, whereas excessive oxidative stress induced by 30 J/cm^2^ irradiation may be detrimental.

Seahorse extracellular flux analysis further demonstrated that 15 J/cm^2^ irradiation enhanced mitochondrial respiratory activity (Fig. 5G), including basal respiration, ATP-linked respiration, maximal respiration and spare respiratory capacity (Fig. 5H). Basal extracellular acidification rate (ECAR) was also moderately increased, suggesting enhanced overall metabolic activity during chondrogenesis (Fig. 5H).

CCO activity was then measured to evaluate mitochondrial respiratory chain complex IV function [23]. The 15 J/cm^2^ group showed increased CCO activity and relative CCO activity compared with the 0 J/cm^2^ and TC groups (Fig. 5I). These results demonstrate that 15 J/cm^2^ 660 nm LED irradiation enhances mitochondrial function, including membrane potential, ATP production, mitochondrial network organization, respiratory activity, and CCO function, while inducing a moderate ROS response.

### 2.5 PI3K/Akt/mTOR/GSK3β–SOX9 signaling mediates the pro-chondrogenic effect of 15 J/cm^2^ irradiation

To identify the signaling pathway linking mitochondrial activation to chondrogenesis, we examined the PI3K/Akt/mTOR/GSK3β-SOX9 axis [24]. Western blotting showed increased phosphorylation ratios of PI3K, Akt, mTOR and GSK3β in the 15 J/cm^2^ group at day 14 (Fig. 6A and B). The increased p-GSK3β/GSK3β ratio was interpreted as enhanced inhibitory phosphorylation of GSK3β, which may reduce its negative regulatory activity and contribute to downstream chondrogenic signaling activation. These changes were less pronounced in the 5 J/cm^2^ group and were not reproduced by the TC group.

**Fig. 6:**
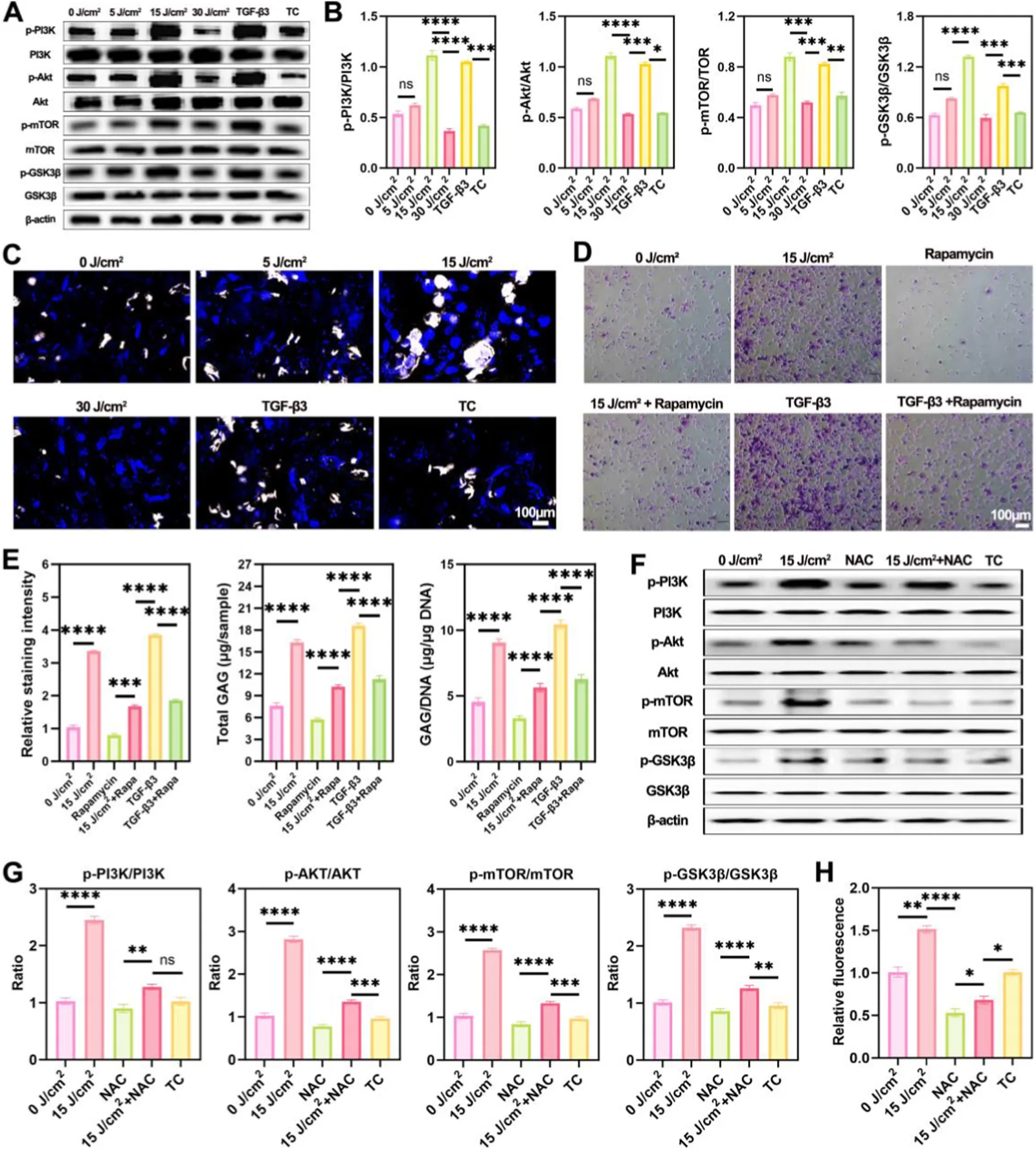
| PI3K/Akt/mTOR/GSK3β–SOX9 signaling mediates the pro-chondrogenic effect of 15 J/cm^2^ 660 nm LED irradiation. **(A)** Representative western blot images of p-PI3K/PI3K, p-Akt/Akt, p-mTOR/mTOR and p-GSK3β/GSK3β in BMSC pellets at day 14 after treatment with 0, 5, 15 or 30 J/cm^2^ 660 nm LED irradiation, TGF-β3 or TC. **(B)** Quantification of the relative phosphorylation ratios of p-PI3K/PI3K, p-Akt/Akt, p-mTOR/mTOR and p-GSK3β/GSK3β in the indicated groups. **(C)** Representative SOX9 immunofluorescence images showing SOX9 nuclear localization in BMSC pellets at day 14 after treatment with 0, 5, 15 or 30 J/cm^2^ 660 nm LED irradiation, TGF-β3 or TC. **(D)** Representative Alcian blue staining images of BMSC pellets in the 0 J/cm^2^, 15 J/cm^2^, rapamycin, 15 J/cm^2^ + rapamycin, TGF-β3 and TGF-β3 + rapamycin groups. For rapamycin-treated groups, BMSC pellets were pretreated with the mTOR inhibitor rapamycin before 660 nm LED irradiation or TGF-β3 stimulation. **(E)** Quantification of Alcian blue staining intensity, total GAG content and GAG/DNA ratio in the indicated groups. **(F)** Representative western blot images and **(G)** corresponding quantification of p-PI3K/PI3K, p-Akt/Akt, p-mTOR/mTOR and p-GSK3β/GSK3β in BMSC pellets in the 0 J/cm^2^, 15 J/cm^2^, NAC, 15 J/cm^2^ + NAC and TC groups. For NAC-treated groups, BMSC pellets were pretreated with NAC before 660 nm LED irradiation or TGF-β3 stimulation. Western blot data were normalized to the corresponding total protein levels and expressed relative to the 0 J/cm^2^ group. **(H)** DCF fluorescence intensity showing intracellular ROS levels in the same groups. Data are presented as mean ± SEM; n = 5 biologically independent samples. Statistical significance was determined by one-way ANOVA with Tukey's multiple-comparison test. ns, not significant; *P < 0.05, **P < 0.01, ***P < 0.001, ****P < 0.0001. (For interpretation of the references to colour in this figure legend, the reader is referred to the Web version of this article.)

SOX9 nuclear localization was then evaluated by immunofluorescence staining. The 15 J/cm^2^ group showed stronger nuclear SOX9 signal than the 0 J/cm^2^ and TC groups (Fig. 6C). Quantitative analysis further confirmed increased nuclear/cytoplasmic SOX9 ratio, nuclear SOX9 intensity and percentage of SOX9-positive nuclei after 15 J/cm^2^ irradiation (Fig. S7). These results indicate that LED-induced pathway activation is associated with SOX9 nuclear activation, a key event in chondrogenic transcriptional regulation.

To test whether PI3K signaling is required for LED-induced chondrogenesis, BMSCs were treated with LY294002 [25]. RT-qPCR showed that LY294002 attenuated the 15 J/cm^2^-induced upregulation of *Sox9*, *Col2a1* and *Acan* (Fig. S8A). The relative chondrogenic gene index was also reduced by LY294002 treatment (Fig. S8B). To further investigate the hierarchical relationship between PI3K, Akt and downstream signaling events, BMSCs were pretreated with the PI3K inhibitor LY294002 or the selective Akt inhibitor MK-2206 before 15 J/cm^2^ 660 nm LED irradiation. Western blot analysis showed that 15 J/cm^2^ LED irradiation markedly increased the phosphorylation of PI3K, Akt, mTOR and GSK3β compared with the control group. LY294002 pretreatment attenuated PI3K phosphorylation and concomitantly reduced the phosphorylation of Akt, mTOR and GSK3β. In contrast, MK-2206 markedly suppressed Akt phosphorylation without significantly affecting PI3K phosphorylation, while simultaneously decreasing the phosphorylation of downstream mTOR and GSK3β. These findings are consistent with Akt acting downstream of PI3K and transmitting LED-induced signaling to the mTOR/GSK3β pathway. Consistently, pharmacological inhibition of either PI3K or Akt markedly reduced the expression of the chondrogenic transcription factor SOX9 and the cartilage matrix-associated proteins COL2A1 and ACAN compared with the LED-treated group (Fig. S9A and B). Furthermore, Alcian blue staining showed that both LY294002 and MK-2206 significantly attenuated the enhanced cartilage matrix deposition induced by 15 J/cm^2^ LED irradiation (Fig. S9C and D). Collectively, these findings support the functional involvement of the PI3K/Akt/mTOR/GSK3β–SOX9 signaling cascade in mediating the pro-chondrogenic effects of 660 nm LED irradiation.

The role of mTOR signaling was further examined using rapamycin [26]. Alcian blue staining showed that rapamycin reduced GAG deposition induced by 15 J/cm^2^ irradiation (Fig. 6D). Quantification of relative staining intensity, total GAG content and GAG/DNA ratio confirmed that mTOR inhibition weakened LED-induced matrix production (Fig. 6E). These data suggest that mTOR signaling is functionally involved in the matrix-promoting effect of 15 J/cm^2^ irradiation.

To determine whether ROS signaling lies upstream of pathway activation, BMSCs were pretreated with N-acetyl-L-cysteine (NAC) [27]. NAC reduced ROS fluorescence and attenuated the phosphorylation of PI3K, Akt, mTOR and GSK3β induced by 15 J/cm^2^ irradiation (Fig. 6F–H). In addition, NAC or mitochondrial inhibition reduced the 15 J/cm^2^-induced increases in Sox9, Col2a1, Acan, GAG/DNA, chondrogenic protein index, SOX9, Aggrecan and COL II levels (Fig. S10A–E). Together, these inhibitor experiments support a mechanistic model in which 660 nm LED irradiation enhances mitochondrial function, induces moderate ROS signaling and activates the PI3K/Akt/mTOR/GSK3β-SOX9 pathway to promote BMSC chondrogenesis (Fig. S11).

This mechanistic framework is important because it provides more than a phenomenological observation of LED-enhanced chondrogenesis. By using ROS scavenging, mitochondrial inhibition, PI3K inhibition, Akt inhibition and mTOR inhibition, the data demonstrate that mitochondrial ROS-dependent signaling is required for the pro-chondrogenic response. Thus, 660 nm LED irradiation acts as a controllable physical stimulus that modulates intracellular metabolic signaling to direct stem cell fate.

### 2.6 15 J/cm^2^ irradiation preserves the chondrogenic phenotype under inflammatory stimulation

Because cartilage repair often occurs in a pro-inflammatory environment, we next established an *in vitro* cartilage pellet inflammatory injury model using interleukin-1β (IL-1β) stimulation (Fig. 7A). RT-qPCR analysis showed that IL-1β markedly increased the expression of inflammatory and matrix-degrading genes, including *Mmp13*, *Adamts5*, *Cox-2*, *Il-6* and *Tnf-α* (Fig. 7B). Treatment with 15 J/cm^2^ irradiation reduced these inflammatory and catabolic markers more effectively than 5 or 30 J/cm^2^ irradiation.

**Fig. 7:**
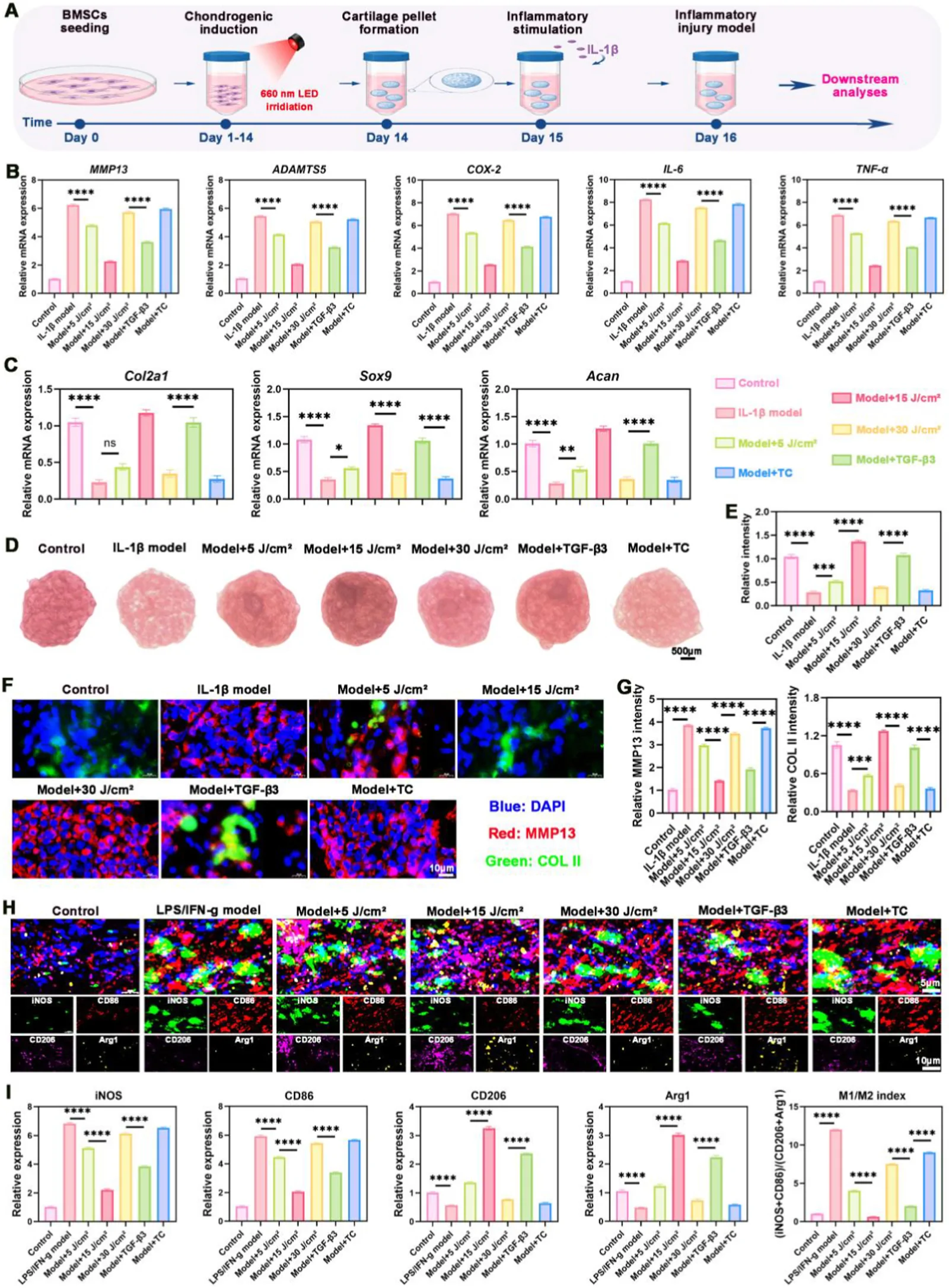
| 15 J/cm^2^ 660 nm LED irradiation preserves the chondrogenic phenotype under inflammatory stimulation. **(A)** Schematic illustration of the *in vitro* cartilage pellet inflammatory injury model. Mature BMSC-derived cartilage pellets were stimulated with IL-1β to establish an inflammatory injury model, followed by histological, molecular and biochemical analyses. **(B)** RT-qPCR analysis of inflammatory and matrix-degrading genes, including *Mmp13*, *Adamts5*, *Cox-2*, *Il-6* and *Tnf-α*, in the Control, IL-1β model, Model + 5 J/cm^2^, Model + 15 J/cm^2^, Model + 30 J/cm^2^, Model + TGF-β3 and Model + TC groups. **(C)** RT-qPCR analysis of chondrogenic genes, including *Sox9*, *Col2a1* and *Acan*, in the indicated groups. **(D)** Representative Safranin O staining images showing GAG deposition in cartilage pellets after IL-1β-induced inflammatory stimulation. **(E)** Quantification of relative Safranin O staining intensity. **(F)** Representative immunofluorescence staining images of COL II and MMP13 in cartilage pellets. **(G)** Quantification of relative COL II and MMP13 fluorescence intensity. **(H)** Representative immunofluorescence staining images of M1 markers (iNOS and CD86) and M2 markers (CD206 and Arg1) in macrophages after LPS/IFN-γ-induced inflammatory stimulation. (I) RT-qPCR analysis of iNOS, CD86, CD206 and Arg1 expression, together with the M1/M2 index, in the Control, LPS/IFN-γ model, Model + 5 J/cm^2^, Model + 15 J/cm^2^, Model + 30 J/cm^2^, Model + TGF-β3 and Model + TC groups. The TC group was temperature-matched to the 30 J/cm^2^ LED group without light exposure. Data are presented as mean ± SEM; n = 5 biologically independent samples. Statistical significance was determined by one-way ANOVA with Tukey's multiple-comparison test for B, C, E, G and I. ns, not significant; *P < 0.05, **P < 0.01, ***P < 0.001, ****P < 0.0001.

In parallel, IL-1β stimulation reduced the expression of chondrogenic genes, including Sox9, Col2a1 and Acan (Fig. 7C). Notably, 15 J/cm^2^ irradiation partially restored these chondrogenic markers under inflammatory stimulation (Fig. 7C). Safranin O staining showed that IL-1β reduced GAG-rich matrix deposition, whereas 15 J/cm^2^ irradiation preserved Safranin O staining intensity (Fig. 7D and E). COL II/MMP13 immunofluorescence further demonstrated that 15 J/cm^2^ irradiation maintained COL II expression and suppressed MMP13 accumulation in inflammatory pellets (Fig. 7F and G).

Macrophage polarization was also examined to evaluate the potential immunomodulatory effect of LED treatment. lipopolysaccharide/interferon-γ (LPS/IFN-γ) stimulation induced an inflammatory M1-like phenotype [28], as indicated by increased iNOS and CD86 expression and decreased CD206 and Arg1 expression (Fig. 7H and I). In contrast, 15 J/cm^2^ treatment reduced M1 marker expression and increased M2 marker expression, leading to a lower M1/M2 index.

These results suggest that 15 J/cm^2^ irradiation not only promotes chondrogenesis under standard induction conditions but also preserves the chondrogenic phenotype under inflammatory stress. This is particularly relevant for tissue repair because inflammatory cytokines can promote matrix degradation and destabilize cartilage-like tissue. The ability of 15 J/cm^2^ irradiation to suppress inflammatory catabolic responses while preserving SOX9, COL II, ACAN and GAG deposition may contribute to improved durability of regenerated cartilage-like tissue *in vivo*.

### 2.7 15 J/cm^2^-preconditioned BMSC–scaffold constructs improve tracheal cartilage defect repair and partially restore native-like structural and functional properties *in vivo*

Finally, we evaluated whether LED-preconditioned BMSC-scaffold constructs could improve tracheal cartilage repair *in vivo*. BMSC-loaded ring-shaped scaffolds were preconditioned with 660 nm LED irradiation for 14 days *in vitro* and then implanted into a tracheal cartilage defect model created by resection of one native tracheal cartilage ring (Fig. 8A). Native tracheal tissue was included as a physiological reference for direct comparison of airway morphology, cartilage matrix composition and mechanical function.

**Fig. 8:**
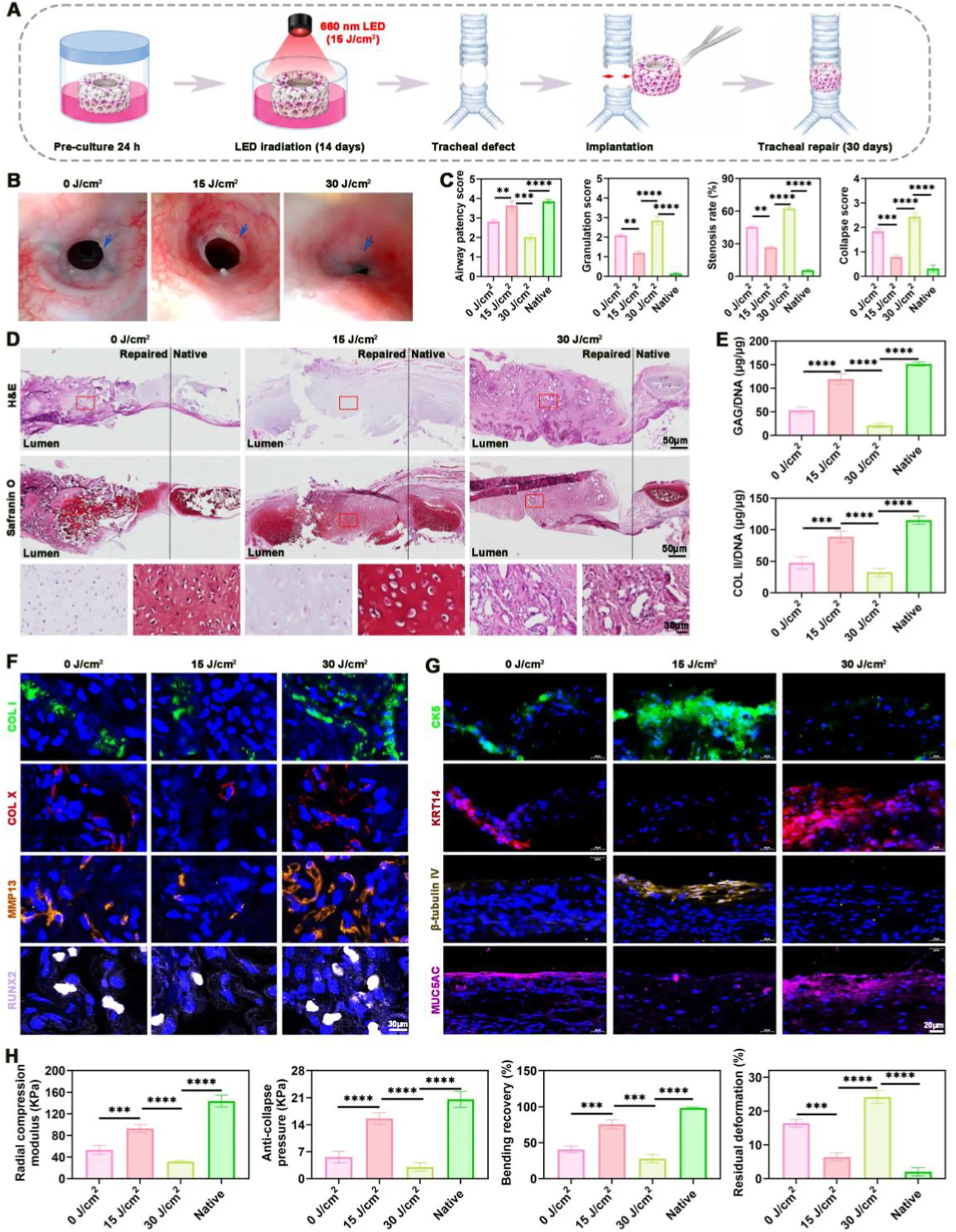
| 15 J/cm^2^-preconditioned BMSC–scaffold constructs improve tracheal cartilage defect repair and partially restore structural and functional properties toward native trachea *in vivo*. **(A)** Schematic illustration of the *in vitro* 660 nm LED preconditioning and implantation procedure. BMSC-loaded ring-shaped porous scaffolds were pre-cultured for 24 h, preconditioned with 660 nm LED irradiation for 14 days, and subsequently implanted into a tracheal cartilage ring defect created by resection of one native tracheal cartilage ring. Repaired tracheal segments were evaluated at day 30 after implantation. **(B)** Representative bronchoscopic images of the repaired trachea at day 30 after implantation in the 0, 15 and 30 J/cm^2^ groups. Blue arrows indicate the anastomotic sites. **(C)** Quantitative bronchoscopic assessment of airway patency, granulation formation, stenosis and airway collapse in the 0, 15 and 30 J/cm^2^ groups, with native trachea included as a physiological reference. **(D)** Representative H&E and Safranin O staining images of repaired tracheal tissues at day 30 in the 0, 15 and 30 J/cm^2^ groups, showing cartilage-like tissue formation and matrix deposition. Black dotted lines outline the repaired zone and adjacent native tracheal tissue. Red boxes indicate the regions magnified in the lower panels. **(E)** Quantification of GAG/DNA and COL II/DNA levels in repaired tracheal tissues from the 0, 15 and 30 J/cm^2^ groups and in native tracheal tissue. **(F)** Representative immunofluorescence staining images of COL I, COL X, RUNX2 and MMP13 in repaired tracheal tissues at day 30 in the indicated groups. **(G)** Representative immunofluorescence staining images of CK5, KRT14, β-tubulin IV and MUC5AC in repaired tracheal tissues at day 30 in the 0, 15 and 30 J/cm^2^ groups, showing airway epithelial regeneration. **(H)** Quantitative comparison of radial compression modulus, anti-collapse pressure, bending recovery and residual deformation among repaired tracheal segments in the 0, 15 and 30 J/cm^2^ groups and native trachea. Native tracheal tissue was included as a physiological benchmark for evaluating the extent of mechanical functional restoration. Data are presented as mean ± SEM; n = 5 biologically independent animals. Statistical significance was determined by Kruskal–Wallis test followed by Dunn's multiple-comparison test for **C**, one-way ANOVA with Tukey's multiple-comparison test for **E** and **H**. **P < 0.01, ***P < 0.001, ****P < 0.0001. (For interpretation of the references to colour in this figure legend, the reader is referred to the Web version of this article.)

At day 30 after implantation, bronchoscopic observation showed improved airway morphology in the 15 J/cm^2^ group compared with the 0 and 30 J/cm^2^ groups (Fig. 8B). Quantitative bronchoscopic analysis demonstrated higher airway patency, reduced granulation formation, lower stenosis and reduced collapse in the 15 J/cm^2^ group (Fig. 8C). Notably, these parameters shifted toward the values observed in native trachea, although complete normalization was not achieved for all indices. These findings indicate that 15 J/cm^2^ preconditioning improved airway-level functional recovery and reduced adverse luminal remodeling after reconstruction.

Histological analysis further demonstrated improved structural restoration of the reconstructed cartilage region. H&E staining revealed more organized tissue architecture in the 15 J/cm^2^ group, whereas Safranin O staining showed more abundant and spatially continuous proteoglycan-rich cartilage-like matrix compared with the 0 and 30 J/cm^2^ groups (Fig. 8D). The adjacent native tracheal cartilage visible within the same sections provided anatomical context for evaluating tissue integration at the repair interface. Consistently, biochemical analysis showed that GAG/DNA and COL II/DNA levels were markedly increased in the 15 J/cm^2^ group compared with the 0 and 30 J/cm^2^ groups and approached, but remained below, those of native tracheal tissue (Fig. 8E). These results demonstrate that optimized LED preconditioning promotes substantial restoration of cartilage-specific extracellular matrix toward the native tracheal phenotype.

Immunofluorescence staining showed that COL I, COL X, RUNX2 and MMP13 expression were reduced in the 15 J/cm^2^ group compared with the other irradiation groups (Fig. 8F and Fig. S12). This suggests that 15 J/cm^2^ preconditioning suppressed fibrotic remodeling, hypertrophic differentiation and matrix degradation *in vivo* [29]. Airway epithelial repair was also improved. CK5 and β-tubulin IV staining indicated restoration of basal epithelial coverage and ciliated epithelial differentiation, whereas KRT14 and MUC5AC expression were reduced [30], suggesting attenuation of injury-associated basal-cell activation and mucus-producing epithelial remodeling (Fig. 8G and Fig. S13). Together, these findings suggest that optimized LED preconditioning supports coordinated regeneration of both cartilage and airway epithelium.

To directly evaluate functional restoration of the reconstructed tracheal ring, we further compared its mechanical properties with those of native trachea. The 15 J/cm^2^ group exhibited a higher radial compression modulus, greater anti-collapse pressure and improved bending recovery, together with lower residual deformation, than the 0 and 30 J/cm^2^ groups (Fig. 8H). Importantly, all four mechanical parameters shifted toward the native tracheal range, although the reconstructed tissues did not fully reach native mechanical performance. This direct comparison indicates that 15 J/cm^2^ LED preconditioning not only enhanced cartilage-like matrix formation but also substantially restored the load-bearing and anti-collapse functions required for maintenance of airway stability.

Overall, the *in vivo* results demonstrate that 15 J/cm^2^ LED preconditioning improves cartilage-like tissue formation, suppresses adverse remodeling, promotes airway epithelial repair and enhances mechanical stability after tracheal cartilage reconstruction. Direct benchmarking against native trachea further shows that the reconstructed tracheal ring acquires structural and functional properties that approach, but do not completely recapitulate, those of normal tracheal tissue. These findings provide stronger evidence for the functional significance of 660 nm LED preconditioning as a strategy to improve BMSC-based tracheal cartilage reconstruction.

## 3 Discussion

In this study, we developed a 660 nm LED preconditioning strategy to enhance the regenerative capacity of BMSCs for tracheal cartilage reconstruction. By systematically optimizing irradiation parameters, we identified 15 J/cm^2^ as an effective condition that improved BMSC proliferation, chondrogenic differentiation, scaffold-based cartilage formation, and *in vivo* tissue repair. These findings indicate that controlled photonic stimulation before implantation can modulate the functional state of BMSCs and provide a potential strategy for improving stem-cell-based cartilage regeneration.

Although photobiomodulation has been increasingly explored for cartilage regeneration and tissue repair, previous studies have mainly investigated irradiation as a direct intervention to modulate local tissue responses, inflammation, or chondrogenic differentiation. For example, 635 nm low-level laser therapy at 20 J/cm^2^ has been reported to improve tracheal wound healing by reducing inflammation and fibrosis while enhancing cartilage regeneration [31]. However, this approach directly irradiated injured tissues to regulate the local repair microenvironment. In contrast, our study applies 660 nm LED irradiation before implantation and introduces a photonic mitochondrial preconditioning strategy to establish a more regenerative BMSC state. The selection of 660 nm LED in this study was based on its well-established mitochondrial photobiomodulatory effects. Red light within this range can regulate mitochondrial function, particularly through CCO-mediated modulation of electron transport, ATP production, and redox signaling. Although 810-850 nm near-infrared light has also been widely investigated, the biological effects of different wavelengths are highly dependent on cell type and irradiation parameters [32]. Therefore, 660 nm was selected as a representative mitochondrial-regulatory stimulus to establish a preconditioned regenerative state in BMSCs, rather than to imply superiority over other wavelengths. Although the irradiation fluences are within a similar range, these studies represent different biological strategies: direct tissue photomodulation versus stem-cell metabolic preconditioning.

Mitochondria have emerged as important regulators of stem-cell fate beyond their classical role in energy production. Previous studies have demonstrated that mitochondrial metabolism influences MSC proliferation, differentiation, and adaptation to environmental stress by regulating energy availability and intracellular signaling networks [33]. In particular, enhanced oxidative phosphorylation has been associated with improved chondrogenic commitment of MSCs, whereas mitochondrial dysfunction may promote senescence and impaired differentiation [34]. Consistent with these findings, our study demonstrates that LED preconditioning can remodel mitochondrial bioenergetic characteristics before transplantation, thereby enhancing the regenerative competence of BMSCs. Unlike previous approaches that mainly modified stem-cell metabolism through biochemical or genetic manipulation, our findings introduce light stimulation as a non-invasive strategy to regulate mitochondrial state and improve the regenerative competence of BMSCs.

Importantly, the optimization of irradiation dosage represents a key aspect of this photonic preconditioning strategy, and the superior biological effect of 15 J/cm^2^ irradiation should not be interpreted as a simple consequence of increased ROS production. Increasing evidence indicates that photobiomodulation exhibits a biphasic dose-response pattern, in which insufficient stimulation fails to activate beneficial cellular responses, whereas excessive stimulation may induce oxidative stress and impair regenerative capacity [35,36]. In this study, 5 J/cm^2^ irradiation induced only limited mitochondrial activation and chondrogenic responses, suggesting that the stimulation intensity was insufficient to fully initiate the regenerative program of BMSCs. In contrast, although 30 J/cm^2^ provided a higher energy input, it did not further enhance chondrogenic outcomes and was accompanied by increased mitochondrial ROS accumulation and oxidative stress-related responses, indicating that excessive irradiation may disrupt cellular redox homeostasis. The optimal effect observed at 15 J/cm^2^ is therefore likely attributed to the establishment of a favorable mitochondrial bioenergetic–redox state rather than simply elevated ROS production. At this dose, LED stimulation enhanced mitochondrial membrane potential, ATP generation, respiratory activity, and CCO function while maintaining controlled redox homeostasis. Therefore, the optimized 15 J/cm^2^ irradiation condition represents a functional window that coordinates mitochondrial activation and cellular adaptation, highlighting the importance of precise photonic modulation rather than simply increasing irradiation intensity.

Beyond its role as an indicator of oxidative status, controlled ROS signaling may function as an active mediator of LED-induced chondrogenesis. ROS are increasingly recognized as signaling molecules that regulate stem-cell differentiation when maintained within an appropriate range, whereas excessive ROS accumulation induces oxidative damage and impairs tissue regeneration [37]. Previous studies have shown that PI3K/Akt/mTOR signaling contributes to MSC survival, proliferation, and chondrogenic differentiation [38]. Importantly, our study goes beyond simply identifying light-induced signaling activation by supporting a model in which mitochondrial bioenergetic remodeling and moderate ROS signaling link 660 nm LED stimulation with PI3K/Akt/mTOR/GSK3β-SOX9-mediated chondrogenesis.

From a tissue engineering perspective, the selection of an appropriate scaffold is also critical for functional tracheal reconstruction [39]. Compared with commonly used hydrogel-based systems, the egg white-derived porous scaffold used in this study provides structural features suitable for cartilage regeneration and tracheal reconstruction. The interconnected porous architecture and mechanical stability of this scaffold support cell adhesion, extracellular matrix deposition, and maintenance of airway structural integrity [40,41]. Although hydrogels offer excellent cell encapsulation and biomimetic microenvironments, their relatively limited mechanical stability may restrict long-term structural maintenance in tubular tissues such as the trachea [42]. In contrast, the ring-shaped porous scaffold provides a stable three-dimensional framework that supports cell attachment, matrix deposition, and airway structural integrity. Therefore, this scaffold represents a complementary biomaterial strategy that integrates cellular support with mechanical reinforcement for tracheal tissue engineering.

There are still limitations. Although direct comparison with native trachea in the present study demonstrated substantial improvement in cartilage matrix restoration and mechanical function after 15 J/cm^2^ LED preconditioning, the reconstructed tissue did not fully recapitulate the structural organization or functional properties of native tracheal cartilage. In addition, the *in vivo* observation period was limited to 30 days. Longer-term studies are therefore warranted to determine whether the reconstructed cartilage continues to mature and whether its matrix organization and mechanical performance can further converge toward those of native trachea over time. In addition, a temperature-matched control specifically corresponding to the optimized 15 J/cm^2^ irradiation condition was not included in the current study. Future studies incorporating such controls will further exclude potential thermal contributions and strengthen the interpretation of LED-induced photobiomodulatory effects. Moreover, although the inhibitor experiments support the involvement of mitochondrial ROS and PI3K/Akt/mTOR signaling, genetic approaches targeting CCO subunits, SOX9 or GSK3β would further strengthen causal interpretation. Furthermore, future work should evaluate whether this preconditioning strategy can be adapted to larger animal models and clinically relevant tracheal reconstruction scenarios.

## 4 Conclusion

In conclusion, this study demonstrates that 660 nm LED irradiation can serve as a photonic mitochondrial programming strategy to enhance BMSC-mediated cartilage regeneration. A defined 15 J/cm^2^ red-light input established a favorable mitochondrial bioenergetic–redox state, characterized by enhanced CCO activity, mitochondrial function, ATP production and moderate ROS signaling. This mitochondrial response activated the PI3K/Akt/mTOR/GSK3β–SOX9 axis, thereby promoting COL2A1, ACAN and GAG-rich matrix formation while suppressing fibrotic, hypertrophic and matrix-degrading phenotypes. When applied to BMSC-loaded ring-shaped porous scaffolds, 15 J/cm^2^ LED preconditioning improved cartilage-like matrix deposition, airway epithelial repair, lumen patency and mechanical support in a tracheal cartilage ring defect model. These findings establish 660 nm LED photobiomodulation as a non-invasive, growth factor-sparing and externally controllable preconditioning approach for functional cartilage tissue engineering.

## 5 Experimental section

### 5.1 Isolation and culture of BMSCs

BMSCs were isolated from bone marrow aspirates of healthy New Zealand white rabbits, which are commonly used for tracheal cartilage repair studies. Briefly, male New Zealand white rabbits weighing 2.5-3.0 kg and aged 12 weeks were used as bone marrow donors. All animal procedures were approved by the Animal Ethics Committee of Shanghai Pulmonary Hospital (approval No. K21-355Y), and were performed in accordance with institutional guidelines for the care and use of laboratory animals. Bone marrow was flushed with sterile phosphate-buffered saline (PBS; Gibco, Thermo Fisher Scientific) and filtered through a 70 μm cell strainer. The cell suspension was centrifuged at 300 × g for 5 min, resuspended in complete culture medium and seeded into tissue culture flasks. BMSCs were cultured in low-glucose Dulbecco's modified Eagle's medium (DMEM-LG; Gibco, Thermo Fisher Scientific) supplemented with 10% fetal bovine serum (FBS; Gibco, Thermo Fisher Scientific) and 1% penicillin–streptomycin (Gibco, Thermo Fisher Scientific) at 37°C in a humidified incubator with 5% CO_2_. Non-adherent cells were removed after 24 h, and the medium was replaced every 2-3 days. Passage 3 BMSCs were used for subsequent experiments unless otherwise stated.

### 5.2 Identification of BMSCs

The morphology of passage 3 BMSCs was observed using an inverted phase-contrast microscope (Olympus). Crystal violet staining was performed to visualize cell morphology and colony-like growth. Briefly, cells were fixed with 4% paraformaldehyde (PFA; Servicebio) for 15 min, stained with 0.1% crystal violet solution (Sigma-Aldrich) for 15 min, washed with PBS and imaged.

For surface marker analysis, BMSCs were collected, washed and incubated with fluorophore-conjugated antibodies against CD29, CD44, CD90, CD105, CD34 and CD45 (BD Biosciences) for 30 min at 4°C in the dark. Flow cytometry was performed using a BD LSRFortessa flow cytometer, and data were analyzed with FlowJo software.

For multilineage differentiation, BMSCs were cultured in adipogenic, osteogenic or chondrogenic induction medium according to the manufacturer's instructions (Cyagen Biosciences). Adipogenic differentiation was evaluated by Oil Red O staining, osteogenic differentiation by Alizarin Red S staining and chondrogenic differentiation by Alcian blue staining. Staining intensity was quantified using ImageJ software.

### 5.3 660 nm LED irradiation system and temperature monitoring

A custom continuous-wave LED irradiation system with a nominal peak wavelength of 660 nm was used for photobiomodulation treatment. The irradiance at the sample surface was calibrated using an optical power meter before each experiment. The power density was set to 50 mW/cm^2^, and the irradiation distance between the LED source and the sample surface was fixed at 10 cm. Energy densities of 5, 15 and 30 J/cm^2^ were delivered by exposure for 100, 300 and 600 s, respectively, according to the equation: energy density (J/cm^2^) = power density (W/cm^2^) × irradiation time (s). The 0 J/cm^2^ group was handled identically but received no light exposure. Irradiation was performed once daily throughout the designated culture period. For the 7-day proliferation experiments, the cumulative fluences were 35, 105 and 210 J/cm^2^ for the 5, 15 and 30 J/cm^2^ groups, respectively. For the 14-day chondrogenic induction and scaffold culture experiments, the cumulative fluences were 70, 210 and 420 J/cm^2^ for the 5, 15 and 30 J/cm^2^ groups, respectively. The TC group was temperature-matched to the 30 J/cm^2^ LED group for 600 s without light exposure. During irradiation, culture medium temperature was continuously monitored using an infrared thermal imaging system to exclude nonspecific thermal effects. Detailed irradiation parameters, including wavelength, power density, single-session energy density, exposure time, irradiation distance, treatment frequency, cumulative fluence and temperature changes, are listed in Table S1.

For temperature monitoring, the culture medium temperature was continuously recorded during 0-10 min irradiation using an infrared thermal imaging system. The maximum temperature increase was calculated as ΔTmax. All irradiation experiments were performed under sterile conditions, and the distance between the LED source and sample surface was kept constant throughout the study.

### 5.4 Cell viability and proliferation assays

For CCK-8 assays, BMSCs were seeded in 96-well plates and treated with 0, 5, 15 or 30 J/cm^2^ 660 nm LED irradiation, transforming growth factor-β3 (TGF-β3) or temperature control. Cell proliferation was evaluated at days 1, 3, 5 and 7 using a CCK-8 assay (Dojindo). CCK-8 solution was added to each well and incubated for 1 h at 37°C. Absorbance at 450 nm was measured using a microplate reader (Thermo Fisher Scientific).

For Ki67 immunofluorescence staining, BMSCs were fixed, permeabilized and blocked with 5% bovine serum albumin (BSA; Sigma-Aldrich). Cells were incubated with anti-Ki67 primary antibody (Abcam) overnight at 4°C, followed by Alexa Fluor-conjugated secondary antibody (Invitrogen, Thermo Fisher Scientific). Nuclei were stained with 4′,6-diamidino-2-phenylindole (DAPI), and Ki67-positive cells were quantified from randomly selected fields.

For EdU incorporation assays, BMSCs were incubated with EdU working solution using an EdU cell proliferation kit (Beyotime) at day 3. Cells were fixed with 4% PFA, permeabilized with 0.3% Triton X-100 and stained according to the manufacturer's protocol. Nuclei were counterstained with DAPI (Sigma-Aldrich). Images were acquired using a fluorescence microscope (Zeiss), and the percentage of EdU-positive cells was quantified using ImageJ.

### 5.5 Cell-cycle analysis

Cell-cycle distribution was analyzed by flow cytometry. BMSCs were collected, washed with PBS and fixed with 70% ethanol at 4°C overnight. After washing, cells were incubated with propidium iodide/RNase staining solution (BD Biosciences) for 30 min in the dark. DNA content was analyzed using a BD flow cytometer, and the percentages of cells in G0/G1, S and G2/M phases were quantified using FlowJo software.

### 5.6 Live/dead staining

Cell viability was evaluated using a Live/Dead staining kit containing calcein-AM and propidium iodide (Invitrogen). BMSCs or BMSC-seeded scaffolds were incubated with staining solution for 20-30 min at 37°C in the dark. Live cells were visualized in green, and dead cells were visualized in red. Images were acquired using a fluorescence microscope, and live-cell percentage was quantified using ImageJ.

### 5.7 Chondrogenic pellet culture

For chondrogenic differentiation, BMSCs were centrifuged in 2 ml conical tubes to form cell pellets. Each pellet contained approximately 5 × 10^5^ cells. Pellets were cultured in basal chondrogenic medium consisting of high-glucose DMEM (Gibco), 1% insulin–transferrin–selenium supplement (ITS; Gibco), 100 nM dexamethasone (Sigma-Aldrich), 50 μg/ml ascorbic acid-2-phosphate (Sigma-Aldrich), 40 μg/ml proline (Sigma-Aldrich) and 1 mM sodium pyruvate (Gibco). Unless otherwise stated, LED-treated groups were cultured in basal chondrogenic medium without exogenous TGF-β3, whereas the TGF-β3 group received 10 ng/ml TGF-β3 (R&D Systems) as a biochemical positive control. Pellets were treated with different doses of 660 nm LED irradiation according to group allocation and cultured for 14 days. Medium was changed every 2 days. At day 14, pellets were collected for gross observation, histological staining, biochemical assays, RT-qPCR, western blotting and immunostaining.

### 5.8 Gross morphology and compactness scoring of BMSC pellets

BMSC pellets were photographed at day 14 using a digital camera. Pellet surface area was measured using ImageJ based on calibrated images. Wet weight was measured immediately after removing excess medium. Pellet compactness was scored by two blinded observers according to pellet integrity, surface smoothness and structural compactness.

### 5.9 Histology and immunohistochemistry of BMSC pellets

BMSC pellets were fixed in 4% PFA, dehydrated, embedded in paraffin and sectioned at 5 μm thickness. Sections were stained with haematoxylin and eosin (H&E), Safranin O and Toluidine blue using standard protocols. For COL II immunohistochemistry, sections were deparaffinized, rehydrated and subjected to antigen retrieval using citrate buffer. Endogenous peroxidase activity was blocked with 3% hydrogen peroxide, and sections were blocked with 5% BSA. Sections were incubated with anti-COL II primary antibody (Abcam) overnight at 4°C, followed by HRP-conjugated secondary antibody. Color development was performed using a DAB substrate kit (Servicebio), and nuclei were counterstained with haematoxylin. Images were acquired using a bright-field microscope (Olympus).

### 5.10 Biochemical quantification of cartilage-like matrix

Total GAG content was measured using a dimethylmethylene blue assay kit (Sigma-Aldrich) according to the manufacturer's instructions. Total collagen content was measured using a hydroxyproline assay kit (Sigma-Aldrich). COL II content was quantified using an ELISA kit specific for COL II (Elabscience). DNA content was measured using a PicoGreen dsDNA assay kit (Invitrogen, Thermo Fisher Scientific). Matrix production was normalized to DNA content, and collagen/DNA, COL II/DNA and GAG/DNA ratios were calculated.

### 5.11 RT-qPCR

Total RNA was extracted from BMSCs, pellets or scaffold constructs using TRIzol reagent (Invitrogen, Thermo Fisher Scientific). RNA concentration and purity were measured using a NanoDrop spectrophotometer (Thermo Fisher Scientific). cDNA was synthesized using a reverse transcription kit (Takara). Quantitative PCR was performed using SYBR Green Master Mix (Bio-Rad) on a real-time PCR system (Applied Biosystems QuantStudio). Relative gene expression was calculated using the 2^−ΔΔCt^ method with Gapdh as the internal reference. Genes analyzed included *Sox9*, *Col2a1*, *Acan*, *Col1a1*, *Col10a1*, *Runx2*, *Mmp13*, *Adamts5*, *Cox-2*, *Il-6*, *Tnf-α*, *iNOS*, *CD86*, *CD206* and *Arg1*. The detailed primer sequences for RT-qPCR are listed in Table S2.

### 5.12 Western blotting

Proteins were extracted using RIPA lysis buffer containing protease and phosphatase inhibitors (Thermo Fisher Scientific). Protein concentration was determined using a BCA protein assay kit (Thermo Fisher Scientific). Equal amounts of protein were separated by SDS-PAGE and transferred onto PVDF membranes (Millipore). Membranes were blocked with 5% BSA for 1 h at room temperature and incubated overnight at 4°C with the following primary antibodies: SOX9 (Cell Signaling Technology, Cat. No. 82630, 1:1000), COL II (Abcam, Cat. No. ab34712, 1:1000), Aggrecan (Abcam, Cat. No. ab3778, 1:1000), COL1A1 (Abcam, Cat. No. ab270993, 1:1000), COL10A1 (Abcam, Cat. No. ab58632, 1:1000), RUNX2 (Cell Signaling Technology, Cat. No. 12556, 1:1000), MMP13 (Abcam, Cat. No. ab39012, 1:1000), phospho-PI3K p85 (Tyr458)/p55 (Tyr199) (Cell Signaling Technology, Cat. No. 4228, 1:1000), PI3K p85 (Cell Signaling Technology, Cat. No. 4257, 1:1000), phospho-Akt (Ser473) (Cell Signaling Technology, Cat. No. 4060, 1:1000), total Akt (Cell Signaling Technology, Cat. No. 4691, 1:1000), phospho-mTOR (Ser2448) (Cell Signaling Technology, Cat. No. 5536, 1:1000), total mTOR (Cell Signaling Technology, Cat. No. 2983, 1:1000), phospho-GSK3β (Ser9) (Cell Signaling Technology, Cat. No. 5558, 1:1000), total GSK3β (Cell Signaling Technology, Cat. No. 9315, 1:1000), GAPDH (Cell Signaling Technology, Cat. No. 5174, 1:5000) and β-actin (Cell Signaling Technology, Cat. No. 4970, 1:5000). After washing, membranes were incubated with HRP-conjugated anti-rabbit or anti-mouse secondary antibodies (Cell Signaling Technology, Cat. Nos. 7074 and 7076, 1:5000) for 1 h at room temperature. Protein bands were visualized using enhanced chemiluminescence reagents (Thermo Fisher Scientific), and band intensity was quantified using ImageJ. Phosphorylated proteins were normalized to their corresponding total protein levels, and other target proteins were normalized to β-actin.

To further validate the regulatory role of the PI3K/Akt/mTOR/GSK3β–SOX9 signaling axis, BMSCs or BMSC pellets were assigned to four groups under the same chondrogenic induction conditions: 0 J/cm^2^ control, 15 J/cm^2^ LED irradiation, 15 J/cm^2^ LED irradiation plus the PI3K inhibitor LY294002, and 15 J/cm^2^ LED irradiation plus the selective Akt inhibitor MK-2206. For inhibitor-treated groups, LY294002 or MK-2206 was administered before LED irradiation according to the pharmacological inhibition protocol described in Section 5.24. Protein expression and phosphorylation levels of PI3K, Akt, mTOR and GSK3β, together with SOX9, COL II and ACAN expression, were subsequently evaluated by western blotting.

### 5.13 Immunofluorescence staining

Samples were fixed with 4% PFA, permeabilized with 0.3% Triton X-100 and blocked with 5% BSA. Samples were incubated with primary antibodies against SOX9, COL II, COL1A1, COL10A1, RUNX2, MMP13, OCN, CK5, KRT14, β-tubulin IV, MUC5AC, iNOS, CD86, CD206 or Arg1 overnight at 4°C. After washing, samples were incubated with Alexa Fluor 594-conjugated secondary antibodies (Invitrogen, Thermo Fisher Scientific). Nuclei were counterstained with DAPI. Images were captured using a confocal microscope (Leica SP8). Fluorescence intensity, positive area and positive cell percentage were quantified using ImageJ. For SOX9 nuclear localization, nuclear/cytoplasmic SOX9 ratio, nuclear SOX9 intensity and percentage of SOX9-positive nuclei were calculated.

### 5.14 Preparation of egg white-derived ring-shaped porous scaffolds

Fresh chicken egg white was collected under sterile conditions and homogenized to obtain a uniform protein solution. The solution was filtered to remove insoluble components and degassed before use. The egg white-derived solution was poured into customized ring-shaped molds and processed by gelation and freeze-drying to generate porous ring-shaped scaffolds. The scaffolds were crosslinked using genipin solution (Sigma-Aldrich), thoroughly washed with sterile PBS to remove residual crosslinker and sterilized by ethylene oxide. Before cell seeding, scaffolds were pre-wetted in complete medium overnight. Gross morphology was photographed, and scaffold microstructure was observed by SEM (Hitachi) after dehydration and gold sputter coating.

### 5.15 BMSC seeding onto ring-shaped porous scaffolds

Sterilized ring-shaped porous scaffolds were placed in low-adhesion culture plates and seeded with BMSCs at a density of approximately 1 × 10^6^ cells per scaffold. Cell suspensions were carefully dropped onto the scaffold surface and allowed to attach for 4 h before additional culture medium was added. BMSC-seeded scaffolds were cultured under standard conditions and treated with 0, 5, 15 or 30 J/cm^2^ 660 nm LED irradiation, TGF-β3 or temperature control. Acellular porous scaffolds without BMSC seeding were used as the Control group in scaffold-related assays.

### 5.16 F-actin staining of BMSC-seeded scaffolds

BMSC-seeded scaffolds were fixed with 4% PFA at day 3, permeabilized with 0.3% Triton X-100 and blocked with 5% BSA. F-actin was stained with phalloidin conjugates (Invitrogen, Thermo Fisher Scientific), and nuclei were counterstained with DAPI. Confocal z-stack images were acquired to assess cell morphology. Cell spreading area, aspect ratio and distribution score were quantified using ImageJ.

### 5.17 *In vitro* mechanical testing of scaffold constructs

Mechanical properties of the scaffold constructs were evaluated at day 14 using a universal testing machine (Instron) equipped with a 10 N load cell. Before testing, samples were equilibrated in PBS at room temperature and maintained in a hydrated state throughout the measurements.

For unconfined compression testing, constructs were placed between two parallel plates, preloaded to 0.01 N, and compressed at a constant rate of 0.5 mm/min until 20% strain was reached. Compressive stress was calculated by normalizing the applied force to the initial loaded area of the construct, and compressive strain was calculated from the change in specimen height relative to the initial height. The compressive modulus was determined from the slope of the linear region of the stress–strain curve between 5% and 15% strain and expressed in kPa.

For three-point bending testing, constructs were positioned on two supports with a span of 6 mm, and a vertical load was applied at the midpoint at a displacement rate of 0.5 mm/min until a displacement of 2 mm was reached. The slope (k) of the linear region of the load–displacement curve was determined, and bending stiffness, expressed as flexural rigidity (EI), was calculated according to the three-point bending relationship: EI = kL^3^/48, where L represents the support span. Bending stiffness was expressed in N·mm^2^.

Radial anti-collapse capacity was evaluated by laterally compressing the ring-shaped constructs between two parallel plates at a constant rate of 0.5 mm/min. Compression was continued until the inner lumen diameter was reduced to 50% of its initial value. The corresponding force at 50% lumen narrowing was converted to radial anti-collapse pressure by normalization to the projected loading area of the construct and expressed in kPa.

### 5.18 Mitochondrial membrane potential analysis

Mitochondrial membrane potential was evaluated using a JC-1 staining kit (Beyotime) at day 14. Samples were incubated with JC-1 working solution for 30 min at 37°C, washed and imaged by fluorescence microscopy. Red fluorescence indicated JC-1 aggregates and high mitochondrial membrane potential, whereas green fluorescence indicated JC-1 monomers and low mitochondrial membrane potential. The JC-1 red/green fluorescence ratio was quantified using ImageJ.

### 5.19 MitoTracker staining

Mitochondrial network morphology was assessed using MitoTracker Red CMXRos (Invitrogen, Thermo Fisher Scientific). Samples were incubated with MitoTracker working solution according to the manufacturer's instructions, washed, fixed and counterstained with DAPI. Confocal images were acquired, and mitochondrial network length, branch points and network score were quantified using ImageJ with mitochondrial network analysis plugins where appropriate.

### 5.20 Measurement of mitochondrial H_2_O_2_, intracellular ROS, MDA and 4-HNE

Mitochondrial H_2_O_2_ levels were detected using MitoPY1 (Tocris Bioscience, Cat. No. 4428), a mitochondria-targeted fluorescent probe for hydrogen peroxide. Briefly, BMSCs or BMSC pellets were washed with serum-free medium and incubated with MitoPY1 working solution at 37°C in the dark for 30 min. After incubation, samples were washed three times with PBS to remove excess probe and immediately imaged using a fluorescence or confocal microscope. Green fluorescence signals were acquired using the FITC/GFP channel, with excitation at 510 nm and emission at 550 nm. Fluorescence intensity was quantified using ImageJ and normalized to the 0 J/cm^2^ group.

For assessment of intracellular ROS in pharmacological inhibition experiments, 2′,7′-dichlorodihydrofluorescein diacetate (DCFH-DA) was used as a general ROS-sensitive fluorescent probe. After the indicated treatments, BMSCs or BMSC pellets were incubated with DCFH-DA working solution at 37°C in the dark according to the manufacturer's instructions. Samples were subsequently washed with PBS and immediately imaged under identical acquisition settings. DCF fluorescence intensity was quantified using ImageJ and expressed relative to the 0 J/cm^2^ control group. This assay was used primarily to evaluate the effect of NAC on LED-induced intracellular ROS accumulation.

To further evaluate oxidative stress and lipid peroxidation, MDA and 4-HNE levels were measured using commercial assay kits according to the manufacturers’ protocols. Samples were collected after the indicated irradiation treatments, and MDA and 4-HNE levels were quantified and normalized according to the corresponding assay specifications.

### 5.21 ATP assay

ATP production was measured using an ATP assay kit based on luciferase activity (Beyotime). Samples were lysed at day 14, and luminescence was measured using a microplate reader. ATP content was normalized to total protein concentration measured by BCA assay. Relative ATP levels were calculated by normalization to the 0 J/cm^2^ group.

### 5.22 Seahorse extracellular flux analysis

Mitochondrial respiratory function was evaluated using a Seahorse XF extracellular flux analyzer. Briefly, BMSCs were seeded into Seahorse XF cell culture microplates and treated according to the indicated experimental groups. Before the assay, the culture medium was replaced with Seahorse XF assay medium supplemented with 10 mM glucose, 1 mM sodium pyruvate and 2 mM glutamine, and cells were equilibrated in a non-CO_2_ incubator at 37°C for 1 h. Oxygen consumption rate (OCR) and ECAR were measured under basal conditions and after sequential injection of oligomycin (1 μM), FCCP (1 μM), and rotenone/antimycin A (0.5 μM each). Basal respiration, ATP-linked respiration, maximal respiration and spare respiratory capacity were calculated according to the manufacturer's instructions. After the Seahorse assay, cells in each well were lysed, and total protein content was measured using a BCA protein assay kit. OCR and ECAR values were normalized to total protein content per well and expressed as pmol/min/μg protein and mpH/min/μg protein, respectively.

### 5.23 Cytochrome *c* oxidase (CCO) activity assay

CCO activity was measured using a CCO assay kit (Sigma-Aldrich) to evaluate mitochondrial respiratory chain complex IV function. Briefly, samples were lysed and prepared according to the manufacturer's instructions. CCO activity was determined by monitoring the oxidation of reduced cytochrome *c* using a microplate reader. Relative CCO activity was normalized to protein content and expressed relative to the 0 J/cm^2^ group.

### 5.24 Pharmacological inhibition experiments

Pharmacological inhibition experiments were performed to investigate the functional involvement of mitochondrial activity, ROS signaling, and the PI3K/Akt/mTOR signaling pathway in 660 nm LED-induced BMSC chondrogenesis. BMSCs or BMSC pellets were treated with the indicated pharmacological modulators according to the experimental design. NAC (5 mM) was used as a ROS scavenger, antimycin A (0.5 μM) was used to transiently inhibit mitochondrial respiratory activity, LY294002 (10 μM) was used to inhibit PI3K signaling, MK-2206 (1 μM) was used to selectively inhibit Akt signaling, and rapamycin (100 nM) was used to inhibit mTOR signaling.

For LED–inhibitor experiments, cells or pellets were pretreated with the corresponding inhibitor for 1 h before 15 J/cm^2^ 660 nm LED irradiation. Irradiation was performed in the continued presence of the inhibitor. Immediately after irradiation, the inhibitor-containing medium was removed, samples were washed with PBS, and fresh inhibitor-free culture medium was added. For experiments involving repeated daily LED irradiation, the same 1 h inhibitor pretreatment was performed before each scheduled irradiation session, followed by removal of the inhibitor after irradiation.

Where indicated, inhibitor-alone groups were included to distinguish the direct effects of pharmacological inhibition from its effects on LED-induced responses. TGF-β3-treated groups were included as biochemical chondrogenic controls, and corresponding TGF-β3 plus inhibitor groups were used to evaluate whether the same signaling pathways also contributed to TGF-β3-induced chondrogenic responses. The inhibitors in these comparator groups were used at the same concentrations as those described above, whereas TGF-β3 was administered according to the chondrogenic induction protocol described in Section 5.7.

LY294002, MK-2206, antimycin A, and rapamycin were dissolved in dimethyl sulfoxide (DMSO), whereas NAC was dissolved in sterile PBS or culture medium. The final DMSO concentration was maintained below 0.1% (v/v), and equivalent vehicle concentrations were included in the corresponding control groups where appropriate. Samples were collected at the indicated experimental endpoints for RT-qPCR, western blotting, biochemical assays, histological staining, and ROS measurements.

### 5.25 *In vitro* cartilage pellet inflammatory injury model

BMSC-derived cartilage pellets were generated by chondrogenic induction for 14 days. Mature cartilage pellets were stimulated with recombinant IL-1β (R&D Systems; 10 ng/ml) for 24 h to establish an *in vitro* inflammatory injury model. Samples were assigned to Control, IL-1β model, Model + 5 J/cm^2^, Model + 15 J/cm^2^, Model + 30 J/cm^2^, Model + TGF-β3 and Model + TC groups. After treatment, pellets were collected for RT-qPCR analysis of *Mmp13*, *Adamts5*, *Cox-2*, *Il-6*, *Tnf-α*, *Sox9*, *Col2a1* and *Acan*. Safranin O staining was performed to assess GAG deposition, and COL II/MMP13 immunofluorescence staining was performed to evaluate preservation of the chondrogenic phenotype and matrix degradation.

### 5.26 Macrophage polarization assay

Rabbit bone marrow-derived macrophages were used for the macrophage polarization assay. Briefly, bone marrow cells were isolated from healthy New Zealand white rabbits, filtered through a 70 μm cell strainer and cultured in complete DMEM supplemented with 10% FBS, 1% penicillin–streptomycin and 20 ng/ml macrophage colony-stimulating factor (M-CSF; PeproTech) for 7 days to induce macrophage differentiation. The medium was changed every 2-3 days. Differentiated adherent macrophages were collected and seeded into culture plates for subsequent experiments. To induce an inflammatory M1-like phenotype, macrophages were stimulated with lipopolysaccharide (LPS; Sigma-Aldrich; 100 ng/ml) and interferon-γ (IFN-γ; PeproTech; 20 ng/ml) for 24 h. Cells were assigned to the Control, LPS/IFN-γ model, Model + 5 J/cm^2^, Model + 15 J/cm^2^, Model + 30 J/cm^2^, Model + TGF-β3 and Model + TC groups. For LED-treated groups, macrophages were directly exposed to 660 nm LED irradiation at the indicated energy densities under the same irradiation parameters used for BMSCs. The TC group was temperature-matched to the 30 J/cm^2^ LED group without light exposure. After treatment, cells were collected for immunofluorescence staining and RT-qPCR analysis. Immunofluorescence staining was performed for M1 markers, including iNOS and CD86, and M2 markers, including CD206 and Arg1. RT-qPCR was performed to quantify iNOS, CD86, CD206 and Arg1 expression. The M1/M2 index was calculated based on the ratio of M1-associated marker expression to M2-associated marker expression.

### 5.27 Animal model of tracheal cartilage ring defect repair

All animal procedures were approved by the Animal Ethics Committee of Shanghai Pulmonary Hospital, Tongji University School of Medicine (approval No. K21-355Y), and were performed in accordance with institutional guidelines for the care and use of laboratory animals. Healthy male New Zealand white rabbits weighing 2.5–3.0 kg and aged 16 weeks were used for the tracheal cartilage defect model. Animals were housed under standard laboratory conditions with free access to food and water and were acclimatized for at least 7 days before surgery. Animals were randomly assigned to experimental groups. BMSC-scaffold constructs were preconditioned with 0, 15 or 30 J/cm^2^ 660 nm LED irradiation for 14 days *in vitro* before implantation.

For the tracheal cartilage defect model, animals were anaesthetized, and the cervical trachea was exposed under sterile surgical conditions. One native tracheal cartilage ring was carefully resected to create a circumferential cartilage ring defect while preserving airway continuity as much as possible. The corresponding ring-shaped BMSC-scaffold construct was implanted into the defect site and secured with microsurgical sutures. The incision was closed layer by layer. Postoperative care included monitoring of respiratory status, wound healing and general condition. Animals were sacrificed at day 30 after implantation for bronchoscopic, histological, biochemical, immunofluorescence and mechanical analyses.

### 5.28 Bronchoscopic evaluation

At day 30 after implantation, airway morphology was evaluated using an endoscopic imaging system. Airway patency, granulation tissue formation, stenosis and collapse were assessed from bronchoscopic images by blinded observers. Quantitative scores were calculated using predefined criteria, and stenosis rate was estimated based on lumen narrowing at the repaired segment. The scoring criteria for airway patency, granulation, stenosis and collapse are provided in Table S3. Each parameter was scored from 0 to 4 by two blinded observers, and the mean score was used for analysis.

### 5.29 Histological analysis of repaired tracheal tissues

Repaired tracheal segments were harvested at day 30, fixed in 4% PFA, decalcified if necessary, embedded in paraffin and sectioned. H&E staining was used to evaluate general tissue morphology and integration. Safranin O staining was used to assess cartilage-like matrix and GAG deposition.

### 5.30 Biochemical analysis of repaired tracheal tissues

Repaired tissue samples were digested for biochemical analysis. GAG content was measured using a dimethylmethylene blue (DMMB) assay, and COL II content was quantified using a COL II ELISA kit. DNA content was measured using a PicoGreen dsDNA assay kit. GAG/DNA and COL II/DNA ratios were calculated to evaluate matrix production normalized to cellular content. Native tracheal tissue was processed in parallel using the same biochemical procedures and served as a physiological reference for comparison with the reconstructed tracheal tissues.

### 5.31 Immunofluorescence analysis of repaired tracheal tissues

Paraffin sections of repaired tracheal tissues were subjected to immunofluorescence staining. COL I, COL X, RUNX2 and MMP13 were detected to assess fibrotic, hypertrophic and matrix-degrading responses. CK5, KRT14, β-tubulin IV and MUC5AC were detected to evaluate airway epithelial repair, basal cell activation, ciliated epithelial differentiation and mucus-producing cell response. Sections were incubated with primary antibodies overnight at 4°C, followed by fluorophore-conjugated secondary antibodies. Nuclei were stained with DAPI. Images were captured using a confocal microscope, and positive areas were quantified using ImageJ.

### 5.32 Mechanical testing of repaired tracheal segments

Functional mechanical properties of the repaired tracheal segments were evaluated at day 30 using a universal testing machine equipped with a 10 N load cell. Repaired tracheal segments were carefully harvested, rinsed with PBS, and maintained in a hydrated state throughout testing. For radial compression testing, each tracheal segment was positioned between two parallel plates and preloaded to 0.01 N. The segment was then compressed at a constant rate of 0.5 mm/min. Radial compression modulus was calculated from the slope of the linear region of the radial stress–strain curve and expressed in kPa. To evaluate resistance to airway collapse, compression was continued until the inner lumen diameter was reduced by 50% of its initial value. The corresponding compressive force was normalized to the loaded area of the tracheal segment to obtain the anti-collapse pressure, expressed in kPa.

For bending recovery testing, each tracheal segment was bent to a displacement equivalent to 50% of its outer diameter and maintained at this displacement for 10 s. After release of the load, the segment was allowed to recover for 60 s, and the residual deformation was recorded. Bending recovery was calculated as: bending recovery (%) = [(applied displacement − residual deformation)/applied displacement] × 100%. Residual deformation was expressed as the percentage of the applied displacement remaining after recovery. Native tracheal segments were tested in parallel using the same mechanical testing procedures and served as physiological references for evaluating the extent of functional mechanical restoration in the reconstructed tracheal segments.

### 5.33 Statistical analysis

All quantitative data are presented as mean ± standard error of the mean (SEM). Statistical analyses were performed using GraphPad Prism software. Each biological replicate represented an independent cell isolation, pellet, scaffold construct or animal sample, depending on the experiment. For image-based quantification, multiple randomly selected microscopic fields from the same sample were averaged before statistical analysis to avoid pseudoreplication. Normality and homogeneity of variance were assessed using the Shapiro-Wilk test and Brown-Forsythe or Levene's test, respectively. Comparisons between two groups were performed using an unpaired two-tailed Student's t-test for normally distributed data or the Mann-Whitney *U* test for non-normally distributed data. Comparisons among multiple groups were performed using one-way ANOVA followed by Tukey's multiple-comparison test. If variances were unequal, Welch's ANOVA followed by Games-Howell's multiple-comparison test was used. Time-course data were analyzed using two-way ANOVA followed by Tukey's or Sidak's multiple-comparison test. Ordinal scoring data were analyzed using the Kruskal-Wallis test followed by Dunn's multiple-comparison test. Exact n values are provided in the corresponding figure legends. P < 0.05 was considered statistically significant. ns, not significant; *P < 0.05; **P < 0.01; ***P < 0.001; ****P < 0.0001.

## CRediT authorship contribution statement

**Zhao An:** Data curation, Conceptualization. **Jiajun Zhang:** Funding acquisition, Formal analysis. **Chao Wang:** Methodology, Investigation. **Yunlang She:** Project administration. **Yong Xu:** Software, Resources. **Haichao Zhou:** Software. **Jiang Xia:** Writing – original draft, Visualization, Validation. **Liang Guo:** Writing – original draft, Visualization. **Minjie Ma:** Writing – review & editing, Writing – original draft. **Lei Zhang:** Writing – review & editing, Writing – original draft, Visualization.

## Declaration of competing interest

The authors declare no conflict of interest.

## Data Availability

Data will be made available on request.

## References

[1] Kharbikar B.N., Mohindra P., Desai T.A. (2022). Biomaterials to enhance stem cell transplantation. Cell Stem Cell.

[2] Luo B., Wang S., Song X., Chen S., Qi Q., Chen W., Deng X., Ni Y., Chu C., Zhou G., Qin X., Lei D., You Z. (2024). An encapsulation-free and hierarchical porous triboelectric scaffold with dynamic hydrophilicity for efficient cartilage regeneration. Adv. Mater..

[3] Nordberg R.C., Bielajew B.J., Takahashi T., Dai S., Hu J.C., Athanasiou K.A. (2024). Recent advancements in cartilage tissue engineering innovation and translation. Nat. Rev. Rheumatol..

[4] Liu Q., Han M., Wu Z., Fu W., Ji J., Liang Q., Tan M., Zhai L., Gao J., Shi D., Jiang Q., Sun Z., Lai Y., Xu Q., Sun Y. (2024). DDX5 inhibits hyaline cartilage fibrosis and degradation in osteoarthritis via alternative splicing and G-quadruplex unwinding. Nat. Aging.

[5] Fan Y., Bian X., Meng X., Li L., Fu L., Zhang Y., Wang L., Zhang Y., Gao D., Guo X., Lammi M.J., Peng G., Sun S. (2024). Unveiling inflammatory and prehypertrophic cell populations as key contributors to knee cartilage degeneration in osteoarthritis using multi-omics data integration. Ann. Rheum. Dis..

[6] Gao W., Chen K., He W., Zhao S., Cui D., Tao C., Xu Y., Xiao X., Feng Q., Xia H. (2023). Synergistic chondrogenesis promotion and arthroscopic articular cartilage restoration via injectable dual-drug-loaded sulfated hyaluronic acid hydrogel for stem cell therapy. Compos. B Eng..

[7] Futrega K., Robey P.G., Klein T.J., Crawford R.W., Doran M.R. (2021). A single day of TGF-β1 exposure activates chondrogenic and hypertrophic differentiation pathways in bone marrow-derived stromal cells. Commun. Biol..

[8] Jackson B.T., Finley L.W.S. (2024). Metabolic regulation of the hallmarks of stem cell biology. Cell Stem Cell.

[9] Chen Y.-S., Tian H.-X., Rong D.-C., Wang L., Chen S., Zeng J., Xu H., Mei J., Wang L.-Y., Liou Y.-L., Zhou H.-H. (2025). ROS homeostasis in cell fate, pathophysiology, and therapeutic interventions. Molecular Biomedicine.

[10] Selestin Raja I., Kim C., Oh N., Park J.-H., Hong S.W., Kang M.S., Mao C., Han D.-W. (2024). Tailoring photobiomodulation to enhance tissue regeneration. Biomaterials.

[11] Maghfour J., Ozog D.M., Mineroff J., Jagdeo J., Kohli I., Lim H.W. (2024). Photobiomodulation CME part I: overview and mechanism of action. J. Am. Acad. Dermatol..

[12] Tong J., Wu X., Wang Z., Li X., Yu Y., Zhang Z., Zheng Z., Huang T., Ma Z. (2025). Photobiomodulation stimulates mitochondrial function and cell proliferation in meniscus-derived stem cells (MeSCs) via activation of TRPV1 channel. Sci. Rep..

[13] Hang N.L.T., Aviña A.E., Chang C.J., Yang T.S. (2025). Photobiomodulation in promoting cartilage regeneration. Int. J. Mol. Sci..

[14] Guo L., Liu X., Wang Y., Yi J., Li J., Xu Y., Cai K., Dai W., Feng Q., Tao B. (2024). Enhancing long-segmental tracheal restoration: a self-repairing hydrogel loaded with chondrocytokines for sutureless anastomosis and cartilage regeneration. Mater. Today Bio.

[15] Chen Z., Bo Q., Wang C., Xu Y., Fei X., Chen R. (2025). Single BMSC-derived cartilage organoids for gradient heterogeneous osteochondral regeneration by leveraging native vascular microenvironment. J. Nanobiotechnol..

[16] Li Y., Xun X., Duan L., Gao E., Li J., Lin L., Li X., He A., Ao H., Xu Y., Xia H. (2025). Cartilage structure-inspired nanofiber-hydrogel composite with robust proliferation and stable chondral lineage-specific differentiation function to orchestrate cartilage regeneration for artificial tracheal construction. Bioact. Mater..

[17] Zheng S., Xu L., Bo Q., Gao E., Zheng E., Xie L., Zhao B., Yi J., Li Y., Xu Y., Wang Y., Tao B. (2025). Xanthohumol bulk-modified polyurethane for tracheal repair: a 'killing two birds with one stone' strategy for tailorable mechanics and durable anti-inflammatory efficacy. Mater. Today Bio.

[18] Xu Y., Dai J., Zhu X., Cao R., Song N., Liu M., Liu X., Zhu J., Pan F., Qin L., Jiang G., Wang H., Yang Y. (2022). Biomimetic trachea engineering via a modular ring strategy based on bone-marrow stem cells and atelocollagen for use in extensive tracheal reconstruction. Adv. Mater..

[19] Gao E., Wang Y., Wang P., Wang Q., Wei Y., Song D., Xu H., Ding J., Xu Y., Xia H., Chen R., Duan L. (2023). C-Shaped cartilage development using Wharton's jelly-derived hydrogels to assemble a highly biomimetic neotrachea for use in circumferential tracheal reconstruction. Adv. Funct. Mater..

[20] Tan S.L., Ahmad T.S., Selvaratnam L., Kamarul T. (2013). Isolation, characterization and the multi-lineage differentiation potential of rabbit bone marrow-derived mesenchymal stem cells. J. Anat..

[21] Liu X., Jiang S., Jiang T., Lan Z., Zhang X., Zhong Z., Wu X., Xu C., Du Y., Zhang S. (2024). Bioenergetic-active exosomes for cartilage regeneration and homeostasis maintenance. Sci. Adv..

[22] Tovar-Ferrero O., Rubio J., Zorzano A., Martínez-Corrales G., Liesa M. (2025). Measuring mitochondrial membrane potential. EMBO J..

[23] Turton N., Cufflin N., Dewsbury M., Fitzpatrick O., Islam R., Watler L.L., McPartland C., Whitelaw S., Connor C., Morris C., Fang J., Gartland O., Holt L., Hargreaves I.P. (2022). The biochemical assessment of mitochondrial respiratory chain disorders. Int. J. Mol. Sci..

[24] Tong J., Subbiah S.K., Rampal S., Ramasamy R., Wu X., You Y., Wang J., Mok P.L. (2024). Effect of 660-nm LED photobiomodulation on the proliferation and chondrogenesis of meniscus-derived stem cells (MeSCs). Sci. Rep..

[25] Ming Z., Vining B., Bagheri-Fam S., Harley V. (2022). SOX9 in organogenesis: shared and unique transcriptional functions. Cell. Mol. Life Sci..

[26] Iezaki T., Horie T., Fukasawa K., Kitabatake M., Nakamura Y., Park G., Onishi Y., Ozaki K., Kanayama T., Hiraiwa M., Kitaguchi Y., Kaneda K., Manabe T., Ishigaki Y., Ohno M., Hinoi E. (2018). Translational control of Sox9 RNA by mTORC1 contributes to skeletogenesis. Stem Cell Rep..

[27] Tangtrongsup S., Kisiday J.D. (2019). Differential effects of the antioxidants N-Acetylcysteine and pyrrolidine dithiocarbamate on mesenchymal stem cell chondrogenesis. Cell. Mol. Bioeng..

[28] Chen S., Saeed A.F.U.H., Liu Q., Jiang Q., Xu H., Xiao G.G., Rao L., Duo Y. (2023). Macrophages in immunoregulation and therapeutics. Signal Transduct. Targeted Ther..

[29] Wang B., Fei X., Yin H.F., Xu X.N., Zhu J.J., Guo Z.Y., Wu J.W., Zhu X.S., Zhang Y., Xu Y., Yang Y., Chen L.S. (2024). Photothermal-controllable microneedles with antitumor, antioxidant, angiogenic, and chondrogenic activities to sequential eliminate tracheal neoplasm and reconstruct tracheal cartilage. Small.

[30] Rock J.R., Onaitis M.W., Rawlins E.L., Lu Y., Clark C.P., Xue Y., Randell S.H., Hogan B.L.M. (2009). Basal cells as stem cells of the mouse trachea and human airway epithelium. Proc. Natl. Acad. Sci..

[31] Lee H.S., Lee Y., Kim H., Oh S.J., Hwang C.W., Kang H.W. (2023). Photomodulative effects of low-level laser therapy on tracheal fenestration developed in in vivo model. J. Photochem. Photobiol., B.

[32] Oliveira S., Ferreira D., Padrão J., Catarino S.O., Monteiro F., Zille A., Rodrigues L.R., Silva F.S., Hinckel B.B., Carvalho Ó., Leal A. (2025). Near-infrared photobiomodulation stimulates viability and cartilage matrix synthesis in human chondrocytes. Sci. Rep..

[33] Yan W., Diao S., Fan Z. (2021). The role and mechanism of mitochondrial functions and energy metabolism in the function regulation of the mesenchymal stem cells. Stem Cell Res. Ther..

[34] Trierweiler C., Hockenjos B., Zatloukal K., Thimme R., Blum H.E., Wagner E.F., Hasselblatt P. (2016). The transcription factor c-JUN/AP-1 promotes HBV-related liver tumorigenesis in mice. Cell Death Differ..

[35] Felician M.C.P., Belotto R., Tardivo J.P., Baptista M.S., Martins W.K. (2023). Photobiomodulation: cellular, molecular, and clinical aspects. J. Photochem. Photobiol., A.

[36] Walski T., Grzeszczuk-Kuć K., Mehl J., Bohara R., Trochanowska-Pauk N., Detyna J., Komorowska M. (2024). Biphasic dose-response and effects of near-infrared photobiomodulation on erythrocytes susceptibility to oxidative stress in vitro. J. Photochem. Photobiol., B.

[37] Sena Laura A., Chandel Navdeep S. (2012). Physiological roles of mitochondrial reactive oxygen species. Mol. Cell.

[38] El-Sayed J.M., Khadrawy S.M., Mohamed H.M., Aly M.S., Khalil A., Sabry D., Mohamed T. (2026). Femtosecond laser and bee venom as promising anti-arthritic treatments: modulation of JAK/STAT and PI3K/AKT/mTOR signaling pathways in vivo. J. Photochem. Photobiol., B.

[39] Gao E., Wang Z., Zhao S., Zhu X., Xu S., Xu Y., Wang Y., Xu N., Tao B. (2024). Core-shell structured nanomembrane with sequential immune and vascular isolation effects followed by chondrogenic induction to promote stable stem cell-based subcutaneous cartilage regeneration in large animals. Chem. Eng. J..

[40] Jia Zihao L.H., Yong X.U., Guangdong Z.H.O.U., Xiaoyan T.A.N., Zhang Wei (2019). Porous scaffold based on egg white for cartilage tissue engineering. Journal of Tissue Engineering and Reconstructive Surgery.

[41] Tao B., Xu T., Yu L., Zhang L., Cao G., Gong N., Zhou G., Xiao K., Huo Y., Xia H. (2025). A simple and low-cost method to develop porous egg white scaffolds with controllable shape for cartilage regeneration. Compos. B Eng..

[42] Mammana M., Bonis A., Verzeletti V., Dell'Amore A., Rea F. (2024). Tracheal tissue engineering: principles and state of the art. Bioengineering.

