# Supplementary material for: 660 nm LED preconditioning programs mitochondrial signaling in BMSCs for functional tracheal cartilage regeneration
Source: Mater Today Bio. 2026 Oct 6;41:103729. doi: 10.1016/j.mtbio.2026.103729 (PMC13676265; doi:10.1016/j.mtbio.2026.103729)
Supplement: Supplementary file 1 [file mmc1.docx]

**Supporting Information**

**660 nm LED preconditioning programs mitochondrial signaling in BMSCs for functional tracheal cartilage regeneration**

*Zhao An^1,a^, Jiajun Zhang^2,a^, Chao Wang^1,a^, Yunlang She^1^, Yong Xu^1^, Haichao Zhou^4^, Jiang Xia^4,*^, Liang Guo^1,*^, Minjie Ma^3,*^, Lei Zhang^1,*^*

^1^ Department of Thoracic Surgery, Shanghai Pulmonary Hospital, Tongji University School of Medicine, Shanghai, China;

^2^ Department of Cardiovascular Surgery, Changhai Hospital, Shanghai, China;

^3^ Department of Thoracic Surgery, The First Hospital of Lanzhou University, Lanzhou, China;

^4^ Department of Orthopedics, Tongji Hospital, School of Medicine, Tongji University School of Medicine, Shanghai, China.

^a^ These authors contributed equally to this work.

* Corresponding author: Dr. Jiang Xia, email: dr_xiajiang@tongji.edu.cn; Dr. Liang Guo, email: guoliang700@tongji.edu.cn; Dr. Minjie Ma, email: maminjie24@sina.com; Dr. Lei Zhang, email: [xiaocha1995@tongji.edu.cn](mailto:xiaocha1995@tongji.edu.cn).

**Figure S1 | Identification of BMSCs.**

**(A)** Representative morphology of passage 3 BMSCs. **(B)** Representative crystal violet staining of passage 3 BMSCs. **(C)** Flow cytometry analysis of BMSC surface markers, including CD29, CD44, CD90, CD105, CD34 and CD45. **(D)** Quantification of marker-positive cells. **(E)** Multilineage differentiation potential of BMSCs, as assessed by Oil Red O staining for adipogenic differentiation, Alizarin Red staining for osteogenic differentiation and Alcian blue staining for chondrogenic differentiation. **(F)** Quantitative analysis of lineage-specific staining intensity. Data are presented as mean ± SEM; n = 5 biologically independent samples. Statistical significance was determined by one-way ANOVA with Tukey’s multiple-comparison test for F. *****P* < 0.0001.

**Figure S2 | Temperature changes in the culture medium during 660 nm LED irradiation.**

**(A)** Culture medium temperature was monitored continuously during 0-10 min irradiation in the indicated groups. **(B)** Quantification of the maximum temperature increase (ΔTmax) in the 0, 5, 15, 30 J/cm^2^ and TC groups. The TC group was temperature-matched to the 30 J/cm^2^ LED group without light exposure. Data are presented as mean ± SEM; n = 5 biologically independent samples. Statistical significance was determined by one-way ANOVA with Tukey’s multiple-comparison test for B. ns, not significant; ****P* < 0.001, *****P* < 0.0001.

**Figure S3 | Cell-cycle distribution of BMSCs after 660 nm LED irradiation.**

**(A)** Flow cytometric analysis of cell-cycle distribution in BMSCs treated with 0, 5, 15 or 30 J/cm^2^ 660 nm LED irradiation, TGF-β3 or temperature control (TC). **(B)** Quantification of the percentage of S-phase cells in the indicated groups. The 0 J/cm^2^ group served as the untreated control; TGF-β3 was used as a positive chondrogenic control, and the TC group was temperature-matched to the 30 J/cm^2^ LED group without light exposure. Data are presented as mean ± SEM; n = 5 biologically independent samples. Statistical significance was determined by one-way ANOVA with Tukey’s multiple-comparison test for B. ***P* < 0.01, ****P* < 0.001.

**Figure S4 | Magnified histological and immunohistochemical images of BMSC pellets after chondrogenic induction.**

Magnified images of H&E, Safranin O, Toluidine blue (TB) and COL II immunohistochemical staining of BMSC pellets at day 14 after treatment with 0, 5, 15 or 30 J/cm^2^ 660 nm LED irradiation, TGF-β3 or TC.

**Figure S5 | Evaluation of osteogenic phenotype in BMSC-loaded porous scaffold constructs after different LED irradiation treatments.**

**(A)** Representative immunofluorescence images showing OCN expression in BMSC-loaded porous scaffold constructs after treatment with different irradiation conditions. Groups included control, 0 J/cm^2^, 5 J/cm^2^, 15 J/cm^2^, 30 J/cm^2^, TGF-β3 and TC groups. **(B)** Quantitative analysis of relative OCN fluorescence intensity. Data are presented as mean ± SEM (n = 5 biologically independent samples). Statistical analysis was performed using one-way ANOVA followed by Tukey’s multiple comparison test. ns, not significant; *****P* < 0.0001.

**Figure S6 | ATP production in BMSCs after 660 nm LED irradiation.**

ATP content and relative ATP levels were measured in BMSCs at day 14 after treatment with 0, 5, 15 or 30 J/cm^2^ 660 nm LED irradiation, TGF-β3 or TC. Relative ATP levels were normalized to the 0 J/cm² group. Data are presented as mean ± SEM; n = 5 biologically independent samples. Statistical significance was determined by one-way ANOVA with Tukey’s multiple-comparison test. **P* < 0.05, ****P* < 0.001, *****P* < 0.0001.

**Figure S7 | Quantitative analysis of SOX9 nuclear localization after 660 nm LED irradiation.**

Quantification of the nuclear/cytoplasmic SOX9 ratio, nuclear SOX9 fluorescence intensity and percentage of SOX9-positive nuclei in BMSC pellets at day 14 after treatment with 0, 5, 15 or 30 J/cm^2^ 660 nm LED irradiation, TGF-β3 or TC. Data are presented as mean ± SEM; n = 5 biologically independent samples. Statistical significance was determined by one-way ANOVA with Tukey’s multiple-comparison test. ns, not significant; **P* < 0.05, ***P* < 0.01, *****P* < 0.0001.

**Figure S8 | LY294002 attenuates 15 J/cm^2^-induced chondrogenic gene expression.**

**(A)** RT-qPCR analysis of *Sox9*, *Col2a1* and *Acan* expression in the 0 J/cm^2^, 15 J/cm^2^, LY294002, 15 J/cm^2^ + LY294002, TGF-β3 and TGF-β3 + LY294002 groups. **(B)** Quantification of the relative chondrogenic gene index based on the combined expression of Sox9, Col2a1 and Acan in the indicated groups. The 0 J/cm^2^ group served as the untreated control, and TGF-β3 was used as a positive chondrogenic control. Data are presented as mean ± SEM; n = 5 biologically independent samples. Statistical significance was determined by one-way ANOVA with Tukey’s multiple-comparison test. ***P* < 0.01, *****P* < 0.0001.

**Figure S9 | PI3K and Akt inhibition further validate the functional involvement of the PI3K/Akt/mTOR/GSK3β-SOX9 signaling axis in 15 J/cm^2^ LED-induced BMSC chondrogenesis.**

**(A)** Western blot analysis of the expression levels of phosphorylated and total PI3K, Akt, mTOR, and GSK3β, as well as SOX9, COL2A1, and ACAN proteins in BMSC pellets under different treatment conditions. The treatment groups included 15 J/cm^2^ 660 nm LED irradiation alone and 15 J/cm^2^ LED irradiation combined with LY294002 or MK-2206 inhibitor treatment. **(B)** Quantitative analysis of the relative protein expression levels. Phosphorylated proteins were normalized to their corresponding total protein levels, while other target proteins were normalized to β-actin. **(C)** Representative images of Alcian blue staining showing cartilage-like matrix formation in BMSC pellets under different treatment conditions. **(D)** Quantification of Alcian blue staining intensity. Data are presented as mean ± SEM (n = 3 biologically independent samples). Statistical analysis was performed using one-way ANOVA followed by Tukey’s multiple comparison test. ns, not significant; ****P* < 0.001, *****P* < 0.0001.

**Figure S10 | NAC and antimycin A pretreatment attenuate the pro-chondrogenic effect of 15 J/cm^2^ irradiation.**

**(A)** Schematic illustration of the experimental design. BMSCs or BMSC pellets were pretreated with N-acetyl-L-cysteine (NAC) or antimycin A for 1 h before 15 J/cm^2^ 660 nm LED irradiation to determine whether ROS signaling and mitochondrial respiratory activity mediate the pro-chondrogenic effect of LED irradiation. After irradiation, the inhibitor-containing medium was removed and replaced with fresh inhibitor-free medium. **(B)** RT-qPCR analysis of chondrogenic genes, including *Sox9*, *Col2a1* and *Acan*, in the indicated groups at day 14. (C) Quantification of GAG/DNA levels at day 14. **(D)** Quantification of the relative chondrogenic protein index at day 14. **(E)** Quantification of relative SOX9, ACAN and COL II protein levels at day 14. The 0 J/cm^2^ group served as the untreated control, and TGF-β3 was used as a positive chondrogenic control. Data are presented as mean ± SEM; n = 5 biologically independent samples. Statistical significance was determined by one-way ANOVA with Tukey’s multiple-comparison test. ns, not significant; **P* < 0.05, ***P* < 0.01, *****P* < 0.0001.

**Figure S11 | Proposed mechanism of 660 nm LED-enhanced BMSC chondrogenesis.**

Schematic illustration of the proposed mechanism by which 660 nm LED irradiation promotes BMSC chondrogenic differentiation. 660 nm LED irradiation enhances mitochondrial function and induces moderate ROS signaling, leading to activation of the PI3K/Akt/mTOR/GSK3β signaling axis and subsequent SOX9 activation. Activated SOX9 further promotes COL2A1 and ACAN expression and GAG-rich extracellular matrix deposition, thereby enhancing cartilage-like matrix formation.

**Figure S12 | Quantification of adverse phenotype markers in repaired tracheal tissues.**

Quantitative analysis of COL I-, COL X-, RUNX2- and MMP13-positive areas in repaired tracheal tissues at day 30 after implantation in the 0, 15 and 30 J/cm^2^ groups. Data are presented as mean ± SEM; n = 5 biologically independent animals. Statistical significance was determined by one-way ANOVA with Tukey’s multiple-comparison test. ***P* < 0.01, *****P* < 0.0001.

**Figure S13 | Quantification of epithelial repair markers in repaired tracheal tissues.**

Quantitative analysis of CK5-, KRT14-, β-tubulin IV- and MUC5AC-positive areas in repaired tracheal tissues at day 30 after implantation in the 0, 15 and 30 J/cm^2^ groups. Data are presented as mean ± SEM; n = 5 biologically independent animals. Statistical significance was determined by one-way ANOVA with Tukey’s multiple-comparison test. **P* < 0.05, *****P* < 0.0001.

**Table S1.** Irradiation parameters for BMSC experiments.

| **Parameter** | **0 J/cm^2^** | **5 J/cm^2^** | **15 J/cm^2^** | **30 J/cm^2^** | **TC** |
| --- | --- | --- | --- | --- | --- |
| Light source | LED off | LED | LED | LED | LED off; temperature-matched |
| Nominal peak wavelength | No light | 660 nm | 660 nm | 660 nm | No light |
| Irradiation mode | No irradiation | Continuous-wave | Continuous-wave | Continuous-wave | No irradiation |
| Power density at sample surface | 0 mW/cm^2^ | 50 mW/cm^2^ | 50 mW/cm^2^ | 50 mW/cm^2^ | 0 mW/cm^2^ |
| Single-session fluence | 0 J/cm^2^ | 5 J/cm^2^ | 15 J/cm^2^ | 30 J/cm^2^ | 0 J/cm^2^ |
| Exposure time per session | 0 s | 100 s | 300 s | 600 s | 600 s, temperature-matched to the 30 J/cm^2^ LED group |
| Irradiation distance | 10 cm, same positioning | 10 cm | 10 cm | 10 cm | 10 cm, same positioning; LED off |
| Treatment frequency | Once daily | Once daily | Once daily | Once daily | Once daily |
| Cumulative fluence for 7-day proliferation experiments | 0 J/cm^2^ | 35 J/cm^2^ | 105 J/cm^2^ | 210 J/cm^2^ | 0 J/cm^2^ |
| Cumulative fluence for 14-day chondrogenic/scaffold experiments | 0 J/cm^2^ | 70 J/cm^2^ | 210 J/cm^2^ | 420 J/cm^2^ | 0 J/cm^2^ |
| Temperature monitoring | Yes | Yes | Yes | Yes | Yes |

Footnote: Energy density was calculated according to the equation: fluence (J/cm^2^) = power density (W/cm^2^) × exposure time (s). The 0 J/cm^2^ group was handled identically but received no light exposure. The TC group was exposed for 600 s without light irradiation and was temperature-matched to the 30 J/cm^2^ LED group. Culture medium temperature was continuously monitored during irradiation.

**Table S2.** RT-qPCR primer sequence.

| **Gene** | **Forward primer 5′–3′** | **Reverse primer 5′–3′** |
| --- | --- | --- |
| *Col1a1* | AGGTGCTGATGGCTCTCCT | GGACCACTTTCACCCTTGT |
| *Col10a1* | TCTGTGAGCTCCATGATTGC | GCAGCATTACGACCCAAGATC |
| *Runx2* | CCGCACGACAACCGCACCAT | CGCTCCGGCCCACAAATCTC |
| *Mmp13* | GATGACCTGTCTGAGGAAGACC | GCATTTCTCGGAGCCTGTCAAC |
| *Adamts5* | CCTGCCCACGATGACTTCAT | GGTAGCCCACTTTGTCCTTG |
| *Cox-2* | CAGGCTGAAATCTACCCTCC | GCTCGGCTTCCAGTATTGAG |
| *Il6* | TTGCCAAGCCTTGTCTGAGAT | TTCTCCCCCAGGGAGTTCAC |
| *Tnf* | ATGAGCACTGAAAGCATGATCCGG | GCAATGATCCCAAAGTAGACCTGCCC |
| *Col2a1* | ACAGCAGGTTCACCTATACCG | CCCACTACCGGTGTGTTC |
| *Sox9* | CACACGTCAAGCGACCCATGAA | TCTTCTCGCTCTCGTTCAGCAG |
| *Acan* | GAGGATGGCTTCCACCAGT | TGGGGTACCTGACAGTCTGA |
| *iNOS* | CCAAGGCCAACCGCGAGAAGATGAC | AGGGTACATGGTGGTGCCGCCAGAC |
| *Cd86* | ACTGCTCATCTATACACGGTTACC | GGAAACGTCGTACAGTTCTGTG |
| *Cd206* | CCTGGTGCTGCTGTTCTCTA | GGCATAGGAGTCCTGGTTCA |
| *Arg1* | CTCCAAGCCAAAGTCCTTAGAG | AGGAGCTGTCATTAGGGACATC |
| *Gapdh* | *CCGCCTGGAGAAAGCTGCTAA* | *ACGACCTGGTCCTCGGTGTA* |

**Table S3.** Bronchoscopic scoring criteria for repaired tracheal segments.

| **Parameter** | **Score 0** | **Score 1** | **Score 2** | **Score 3** | **Score 4** |
| --- | --- | --- | --- | --- | --- |
| Airway patency | Complete/near-complete obstruction; <25% lumen patency | Severe compromise; 25–49% lumen patency | Moderate compromise; 50–74% lumen patency | Mild compromise; 75–94% lumen patency | Nearly complete/complete patency; ≥95% lumen patency |
| Granulation formation | No visible granulation tissue | Minimal focal granulation; <10% of repaired region | Mild granulation; 10–25% of repaired region | Moderate granulation; 26–50% of repaired region | Marked/exuberant granulation; >50% of repaired region or obstructive growth |
| Stenosis | No stenosis or <5% lumen narrowing | Mild stenosis; 5–25% lumen narrowing | Moderate stenosis; 26–50% lumen narrowing | Severe stenosis; 51–75% lumen narrowing | Critical stenosis; >75% lumen narrowing or near occlusion |
| Airway collapse | No collapse; <5% dynamic lumen narrowing | Mild collapse; 5–25% dynamic lumen narrowing | Moderate collapse; 26–50% dynamic lumen narrowing | Severe collapse; 51–75% dynamic lumen narrowing | Near-complete/complete collapse; >75% dynamic lumen narrowing |

Note: Lumen patency, stenosis and collapse were estimated relative to the adjacent normal tracheal segment. For airway patency, a higher score indicates better repair; for granulation formation, stenosis and collapse, a lower score indicates better repair. Bronchoscopic images were scored independently by two blinded observers, and the mean value was used for statistical analysis.
